# Endometrial and Menstrual Blood Mesenchymal Stem/Stromal Cells: Biological Properties and Clinical Application

**DOI:** 10.3389/fcell.2020.00497

**Published:** 2020-07-09

**Authors:** Mahmood Bozorgmehr, Shanti Gurung, Saeedeh Darzi, Shohreh Nikoo, Somaieh Kazemnejad, Amir-Hassan Zarnani, Caroline E. Gargett

**Affiliations:** ^1^Reproductive Immunology Research Center, Avicenna Research Institute, Academic Center for Education, Culture and Research (ACECR), Tehran, Iran; ^2^Oncopathology Research Center, Iran University of Medical Sciences, Tehran, Iran; ^3^Centre for Reproductive Health, Hudson Institute of Medical Research, Melbourne, VIC, Australia; ^4^The Ritchie Centre, Hudson Institute of Medical Research, Melbourne, VIC, Australia; ^5^Department of Obstetrics and Gynaecology, Monash University, Melbourne, VIC, Australia; ^6^Immunology Research Center, Iran University of Medical Sciences, Tehran, Iran; ^7^Nanobitechnology Research Center, Avicenna Research Institute, Academic Center for Education, Culture and Research (ACECR), Tehran, Iran; ^8^Department of Immunology, School of Public Health, Tehran University of Medical Sciences, Tehran, Iran

**Keywords:** endometrium, menstrual blood, culture expansion, perivascular MSC, eMSC, MenSC, cell therapy, immunomodulation

## Abstract

A highly proliferative mesenchymal stem/stromal cell (MSC) population was recently discovered in the dynamic, cyclically regenerating human endometrium as clonogenic stromal cells that fulfilled the International Society for Cellular Therapy (ISCT) criteria. Specific surface markers enriching for clonogenic endometrial MSC (eMSC), CD140b and CD146 co-expression, and the single marker SUSD2, showed their perivascular identity in the endometrium, including the layer which sheds during menstruation. Indeed, cells with MSC properties have been identified in menstrual fluid and commonly termed menstrual blood stem/stromal cells (MenSC). MenSC are generally retrieved from menstrual fluid as plastic adherent cells, similar to bone marrow MSC (bmMSC). While eMSC and MenSC share several biological features with bmMSC, they also show some differences in immunophenotype, proliferation and differentiation capacities. Here we review the phenotype and functions of eMSC and MenSC, with a focus on recent studies. Similar to other MSC, eMSC and MenSC exert immunomodulatory and anti-inflammatory impacts on key cells of the innate and adaptive immune system. These include macrophages, T cells and NK cells, both *in vitro* and in small and large animal models. These properties suggest eMSC and MenSC as additional sources of MSC for cell therapies in regenerative medicine as well as immune-mediated disorders and inflammatory diseases. Their easy acquisition via an office-based biopsy or collected from menstrual effluent makes eMSC and MenSC attractive sources of MSC for clinical applications. In preparation for clinical translation, a serum-free culture protocol was established for eMSC which includes a small molecule TGFβ receptor inhibitor that prevents spontaneous differentiation, apoptosis, senescence, maintains the clonogenic SUSD2^+^ population and enhances their potency, suggesting potential for cell-therapies and regenerative medicine. However, standardization of MenSC isolation protocols and culture conditions are major issues requiring further research to maximize their potential for clinical application. Future research will also address crucial safety aspects of eMSC and MenSC to ensure these protocols produce cell products free from tumorigenicity and toxicity. Although a wealth of data on the biological properties of eMSC and MenSC has recently been published, it will be important to address their mechanism of action in preclinical models of human disease.

## Introduction

**FIGURE 1:**
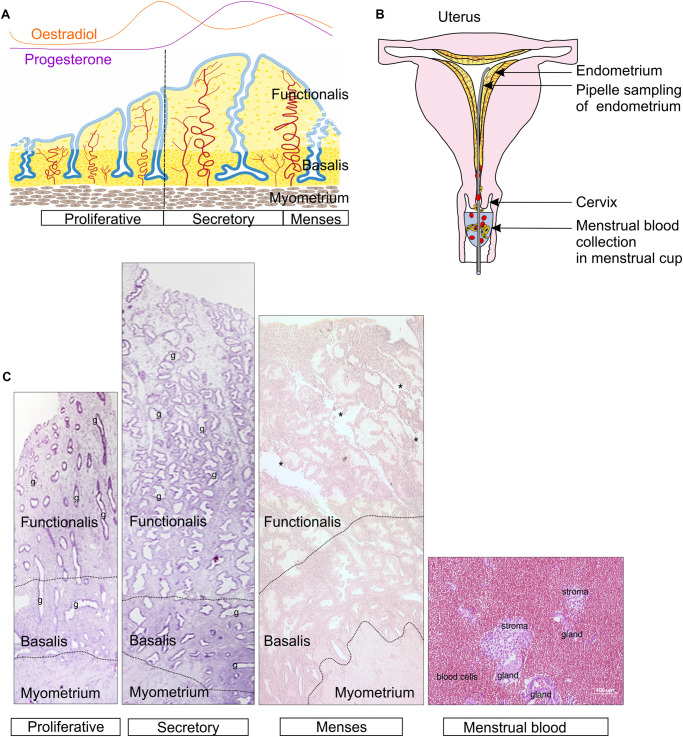
Ovarian and Menstrual cycle, structure of human endometrium and its shedding during menstruation. **(A)** Schematic showing human ovarian hormonal changes corresponding with endometrial growth, differentiation and shedding, during the menstruation, proliferative and secretory phases, respectively, of a menstrual cycle. **(B)** Schematic showing endometrial tissue collection from human endometrium as an office-based procedure using a Pipelle endometrial suction catheter and menstrual fluid using a menstrual cup. **(C)** Histological sections stained with H&E of pre-menopausal human endometrium during the growth (proliferative), differentiation (secretory) and menses stages of the menstrual cycle showing the functionalis, basalis and myometrial layers of the endometrium. *Endometrial tissue breakdown early in the menstrual stage, leaving behind an intact basalis layer. Menstrual blood showing endometrial tissue fragments comprising endometrial stroma, glands and blood cells. Dotted lines define the layers of the endometrium. g, glands. Reproduced with permission from Figure 1A from Elsevier (Gargett et al., 2008) and Figure 1C from Oxford University Press (Nguyen et al., 2012).

Almost all human tissues contain a small resident population of perivascular mesenchymal stem/stromal cells (MSC) (Crisan et al., 2008). MSC have also been identified in a wide variety of small and large mammalian species (Rozemuller et al., 2010), although these have been studied to a lesser degree. Here we discuss a novel source of MSC from the highly regenerative endometrial lining of the uterus (Figure 1A), accessible by biopsy in an office-based procedure without an anesthetic, or non-invasively from menstrual blood (Figure 1B) (Ulrich et al., 2013). This review will focus on the biological properties and recent functional characterization of endometrial MSC (eMSC) and menstrual blood MSC (MenSC). The reader is referred to a recent comprehensive review providing greater details on earlier studies describing the identification and early characterization of endometrial MSC, MenSC and endometrial stromal fibroblasts (Gargett et al., 2016). Both epithelial progenitors and MSC have been identified in human and mouse endometrium, however, it is the endometrial and menstrual blood MSC that comprise the topic of this review.

Recently the MSC field has been criticized, due to the poor characterization of MSC from various sources, which has resulted in underwhelming outcomes of many clinical trials using MSC (Sipp et al., 2018). Counter arguments reiterate the importance of using the appropriate definition of the cells under study (i.e., perivascular MSC versus fibroblasts) (Galipeau et al., 2019). Integrated transcriptomic profiling of human MSC from different tissues show their segregation by tissue of origin (Roson-Burgo et al., 2016; Menard et al., 2020) and distinct tissue-specific MSC immune signatures that are similar between fresh and cultured MSC from the same tissue source (Menard et al., 2020). Herein we will distinguish between perivascular eMSC and endometrial stromal fibroblasts. We will also differentiate between potential regenerative “stem cell” properties and immunomodulatory function of both eMSC and MenSC. It has become clear that the regenerative properties of MSC are mainly due to the trophic factors they secrete. These stimulate endogenous cells to repair damaged tissues, rather than MSC functioning as true stem cells (Bianco et al., 2013). MSC are reparative rather than regenerative. It is also recognized that MSC have profound immunomodulatory effects on innate and adaptive immune cells that promote healing by reducing inflammation and immune responses (Galipeau and Sensebe, 2018). In this review, we recognize these important developments in the MSC field and our review on endometrial and menstrual blood MSC and fibroblasts has been structured around these themes.

Human endometrium is a dynamic remodeling tissue, undergoing cycles of growth, differentiation and shedding on a monthly basis as part of the menstrual cycle (Gargett et al., 2012). These dynamic processes occur about 400 times in women until menopause (Jabbour et al., 2006). During menstruation, the upper functional layer of endometrial tissue sloughs off and the tissue fragments exit the body in menstrual blood, leaving a residual 1–2 mm of endometrial tissue (basalis) overlying the myometrium (uterine muscle) (Figures 1A–C). The raw surface rapidly reepithelializes and the new functional layer (functionalis), comprising epithelial-lined glands and an extensive vascularized stroma regenerates under the influence of rising, circulating estrogen levels (Gargett et al., 2008) in the next cycle. Atrophic postmenopausal endometrium, which transcriptionally resembles cycling basalis endometrium (Nguyen et al., 2012), also regenerates a functionalis-like layer when women take estrogen-only hormone replacement therapy. MSC can be harvested from this regenerated tissue (Ulrich et al., 2014b).

## Human Endometrial MSC

Endometrial MSC were first identified as clonogenic stromal cells, comprising 1.3% of stromal fibroblasts harvested from hysterectomy tissue, which contains both functionalis and basalis endometrium (Chan et al., 2004). The large stromal colonies, comprised densely packed small cells with a fibroblast-like morphology and distinguishable from self-limiting small, sparsely packed colonies, likely originated from colony forming units-fibroblast (CFU-F) and do not vary in frequency during the menstrual cycle. This and their presence in postmenopausal endometrium indicate hormone independence of endometrial CFU-F (Schwab et al., 2005; Ulrich et al., 2014b). In comparison to single small colonies, individual large CFU-F showed high proliferative capacity, undergoing 30 population doublings (PD) and producing > 600 billion cells (Gargett et al., 2009). Single large endometrial CFU-F underwent self-renewal *in vitro* by serial cloning at very low seeding densities (5–10 cells/cm^2^) and differentiated into adipocytes, chondrocytes, myocytes and osteocytes (Gargett et al., 2009). They also expressed the classic pattern of International Society for Cellular Therapies (ISCT) markers (Table 1). These properties indicate that human endometrium contains a small population of MSC.

**TABLE 1:** Comparison of phenotypic markers of endometrial, menstrual, bone marrow, and adipose tissue MSC isolated by plastic adherence or by SUSD2 or CD34 cell sorting.

	Markers (% positive cells)															
MSC type	CD29	CD31	CD34	CD44	CD45	CD73	CD90	CD105	CD140b	CD146	SUSD2	STRO1	SSEA4	OCT4	HLA-DR	References
Pericyte CD146^+^CD140b^+^ eMSC in SFM	95.1	–	0	86	0.8	79	–	92	69	37	42.9	–	–	–	–	Rajaraman et al., 2013
Perivascular SUSD2^+^ eMSC, freshly isolated	11.6	5.3	–	77	4.7	99	71	99	85	28	95	60	–	–	–	Masuda et al., 2012
Perivascular SUSD2^+^ eMSC in SM	93	2.3	0.5	93	–	99.9	98	99.5	50	1–2	–	0.9	–	–	0	Darzi et al., 2016
Perivascular SUSD2^+^ eMSC in SFM	–	–	–	-	–	–	–	–	53	1	69	–	–	–	–	Gurung et al., 2015
Perivascular SUSD2^+^ bmMSC in SM	100	< 1	0	–	0	100	98	100	100	–	–	–	–	–	–	Sivasubramaniyan et al., 2013
Perivascular SUSD2^+^ bmMSC, freshly isolated	–	26	5	70	90	–	0	20	–	4	–	–	–	–	22	Busser et al., 2015
Perivascular SUSD2^+^ bmMSC in SM	–	5.3	1.4	83	1.6	–	81	64	–	95	–	–	–	–	1.7	Busser et al., 2015
CD34^+^ adMSC, freshly isolated	–	38	78	81	57	–	39	28	–	41	–	–	–	–	10	Busser et al., 2015
CD34^+^ adMSC in SM	–	6.7	17	98	5.8	–	56	79	–	13	–	–	–	–	4.9	Busser et al., 2015
Plastic adherent, passaged, CD117^+^ MenSC in SM	65	–	5.7	99.8	2.1	–	99.8	99.8	–	–	–	–	90	–	1.6	Patel et al., 2008
Plastic adherent MenSC	99.6	–	0.7	99.4	0.8	99.2	93.1	99.6	–	74.9	–	5.6	–	97.5	0	Musina et al., 2008
Plastic adherent bmMSC	> 95	–	0	97.8	0	≥ 90	≥ 90	≥ 90	≥ 95	15–20	–	3.8	–	0	0	Vogel et al., 2003; Musina et al., 2008; Bourin et al., 2013
Plastic adherent adMSC	>95	–	0	64	0	25	55	5	–	21	–	0	–	–	1	Gronthos et al., 2001; Mitchell et al., 2006

*Adapted from Darzi et al. Stem Cells Translational Medicine 2016 (Darzi et al., 2016) with permission from John Wiley and Sons Publishers. adMSC, Adipose derived MSC; bmMSC, Bone marrow MSC; HLA-DR, Human Leukocyte Antigen DR; MenSC, Menstrual blood MSC; MSC, Mesenchymal Stem/Stromal cell; SFM, DMEM/F12 Serum Free Medium supplemented with EGF and FGF2; SM, DMEM/F12 serum medium contains 10% FBS; –, no data.*

### Markers of eMSC

**FIGURE 2:**
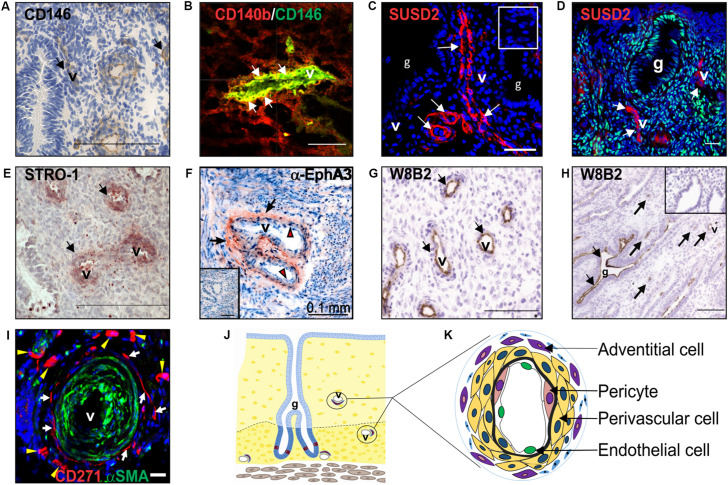
Clonogenic endometrial MSC identified using various surface markers. Surface markers **(A)** CD146, **(B)** co-expression of CD140b/CD146, SUSD2 (red) in **(C)** pre-menopausal and **(D)** postmenopausal endometrium, showing no colocalization with ERα (green), **(E)** STRO-1 and **(F)** EphA3 (red, black arrows), mark clonogenic perivascular eMSC. **(G)** MSCA-1 (TNAP) detected by the W8B2 antibody is expressed by both perivascular and **(H)** epithelial cells in the human endometrium**. (I)** CD271 (red) adventitial perivascular cells mark clonogenic cells in ovine endometrium. **(J)** Schematic of endometrium showing location of endometrial MSC around the vessels. **(K)** Schematic enlargement of blood vessels from **(J)** showing different vascular cell types in the endometrium. Human CD140b^+^CD146^+^ cells are pericytes (pink cytoplasm), and SUSD2, STRO-1, and EphA3 cells are perivascular cells (yellow cytoplasm). Ovine CD27^+^ cells are adventitial cells (violet cytoplasm). Mouse CD34^+^KLF4^+^ and LRC cells are also represented by perivascular cells (yellow cytoplasm). Images reprinted with permission from: **(A,E)** Figures 1E, **4C** from Oxford University Press (Schwab et al., 2008), **(B)** **Figure 4I** from Oxford University Press (Schwab and Gargett, 2007), **(C)** Figure 1A from Bioscientifica (Yang et al., 2019), **(D)** **Figure 4A** from Oxford University Press (Ulrich et al., 2014b), **(F)** Figure 1A from Public Library of Science (To et al., 2014), **(G,H)** **Figures 5C,D** from Mary Ann Liebert (Sobiesiak et al., 2010), **(I)** **Figure 5D** from Public Library of Science (Letouzey et al., 2015) and **(K)** Figure 2 from Wiley Online Library (Darzi et al., 2016).

eMSC were identified as perivascular cells by comparing cloning efficiencies of endometrial stromal cells purified using flow cytometry for several surface markers used for bone marrow MSC (Figure 2A) (Schwab et al., 2008). Most CFU-F were from cells co-expressing CD140b and CD146 (Schwab and Gargett, 2007). CD140b^+^CD146^+^ cells, comprising 1.5% of endometrial stromal cells, were enriched eightfold for CFU-F over unsorted stromal cells and fulfilled the ISCT MSC criteria (Dominici et al., 2006). Their perivascular identity was revealed in both the basalis and functionalis (Figures 2B,J), indicating that CD140b^+^CD146^+^ eMSC could be isolated from endometrial biopsies and would be shed in menstrual blood (Darzi et al., 2016). Gene profiling CD14b^+^CD146^+^ eMSC versus CD140b^+^CD146^–^ endometrial fibroblasts showed 762 differentially expressed genes (Spitzer et al., 2012), indicating that perivascular eMSC are distinct from endometrial fibroblasts as for other MSC types (Menard et al., 2020).

Similar to bone marrow MSC, perivascular eMSC are also MSCA-1^+^ (Sobiesiak et al., 2010), a marker antibody that identifies tissue non-specific aminopeptidase (TNAP), however, this marker is not useful for sorting eMSC as it also marks glandular epithelial cells (Figures 2G,H).

To identify a single marker of perivascular eMSC, endometrial stromal cell suspensions were screened with a panel of perivascular and other novel antibodies by flow cytometry and immunohistochemistry of human endometrium (Masuda et al., 2012). Using this strategy, the W5C5 antibody identified a robust marker of stromal cells from pre- and postmenopausal endometrium with MSC properties, enriching clonogenic cells 18-fold over W5C5^–^ fibroblasts (Masuda et al., 2012; Ulrich et al., 2014b). Sushi Domain-containing 2 (SUSD2) was the antigen identified by the W5C5 antibody (Sivasubramaniyan et al., 2013). SUSD2^+^ cells comprise 4.1% of endometrial stromal cells and in addition to satisfying the ISCT criteria, they reconstituted stromal tissue *in vivo* under the kidney capsule of NOD-Scid γ (NSG) mice. Non-ISCT markers also expressed by freshly isolated SUSD2^+^ eMSC include CD117, CD140b, CD146, and STRO-1 (Figure 2E). More clonogenic cells were present in the SUSD2^+^CD146^+^ and SUSD2^hi^ subpopulations than in the CD140b^+^CD146^+^ co-expressing population (Masuda et al., 2012). SUSD2 enables prospective isolation of eMSC from freshly isolated cell suspensions using magnetic bead sorting, providing a more clonogenic population than obtained by flow cytometry sorting, which adversely affects cell viability (Masuda et al., 2012). This is an important consideration for clinical translation.

The specific markers of eMSC show that these cells are located around blood vessels in both the functionalis (Figures 1, 2) indicating they are shed into menstrual fluid as the functionalis breaks down during menstruation (Figure 1B). Similarly, stromal fibroblasts are shed into menstrual fluid. Both eMSC and stromal fibroblasts (MenSC) are shed in numbers proportionate to their composition in endometrial functionalis tissue, with eMSC comprising a minority subpopulation. The adult stem cell properties of human eMSC suggest that stromal fibroblasts are their progeny, and to date the only evidence comes from xenografting SUSD2^+^ eMSC into immunocompromised mice where stromal tissue was generated (Masuda et al., 2012).

### Differentiation of eMSC

Physiologically, eMSC around spiral arterioles differentiate into decidual cells under influence of the pregnancy hormone, progesterone, during the secretory stage of the menstrual cycle (Gellersen and Brosens, 2014). This decidual differentiation spreads to the stromal fibroblasts beneath the luminal epithelium. Decidual cells are specialized secretory cells that provide an immunoprivileged environment for an implanting embryo to establish the materno-fetal interface. Subpopulations of eMSC and stromal fibroblasts undergo senescence during the differentiation process (Lucas et al., 2016) and when no embryo implants, progesterone levels fall and menstruation ensues (Figure 1).

Transcriptional profiling of endometrial SUSD2^+^ eMSC and SUSD2^–^ stromal fibroblasts revealed a distinct gene signature for both cell types following decidual differentiation *in vitro* (Murakami et al., 2014). Known and novel perivascular genes were upregulated in SUSD2^+^ eMSC, which produced lower levels of inflammatory mediators and chemokines *in vitro* compared to SUSD2^–^ stromal fibroblasts. Similarly, the inflammatory gene signature of freshly isolated and cultured CD140b^+^CD146^+^ eMSC had fewer transcripts than CD140b^+^CD146^–^ endometrial stromal fibroblasts (Barragan et al., 2016). Upon decidualization (differentiation) induction SUSD2^+^ eMSC and SUSD2^–^ stromal fibroblasts showed greater divergence of their respective secretomes, with the eMSC producing much higher levels of leukemia inhibitory factor and the chemokine CCL7 than stromal fibroblasts. These varying features highlight differences between perivascular eMSC and stromal fibroblasts.

Embryologically, endometrium derives from the mesoderm. Thus, it is not unexpected that endometrial MSC and stromal fibroblasts can be induced to differentiate into mesodermal lineages. Differentiation of eMSC into classic mesodermal lineages *in vitro* as recommended by the ISCT has been shown for clonogenic endometrial stromal cells, SUSD2^+^ and CD140b^+^CD146^+^ cells (Schwab and Gargett, 2007; Gargett et al., 2009; Masuda et al., 2012; Su et al., 2014). Endometrial stromal fibroblasts also show similar mesodermal lineage differentiation, as do bone marrow-derived stromal cells (Haniffa et al., 2009). The reader is referred to table III in Gargett et al. (2016) for a comprehensive list of studies describing mesodermal lineage differentiation of eMSC and endometrial stromal fibroblasts. Differentiation into endodermal lineages include hepatocytes from clonogenic eMSC *in vitro* (Yang et al., 2014) and insulin-and glucagon-secreting pancreatic lineages both *in vitro* and *in vivo* from endometrial stromal fibroblasts (Li et al., 2010; Santamaria et al., 2011). Similarly, cultured endometrial fibroblasts have been differentiated into ectodermal lineages such as dopamine-secreting neurons *in vitro* and *in vivo*, where they may have also induced endogenous neural cell secretion (Wolff et al., 2011). Oligodendrocyte progenitor cells have been differentiated from endometrial stromal fibroblasts (Ebrahimi-Barough et al., 2013). It is not known if eMSC differentiate into ectodermal lineages.

### Endometrial MSC in Other Species

The dynamic nature of the endometrium is not limited to humans. Endometrium of other species also undergo proliferation and differentiation, and endure high levels of regeneration during their reproductive life (Rozemuller et al., 2010; Lara et al., 2018). No animal models can replace humans, however, they provide preclinical models bridging the gap between the *in vitro* potential of MSC and future clinical use in humans. The identification of endometrial stromal/stem cells in non-human species has made this possible. Mammalian MSC have been identified in the endometrium (Lara et al., 2018) of mice (Chan and Gargett, 2006; Cervello et al., 2007), guinea pig (Ordener et al., 1993), pig (Miernik and Karasinski, 2012; Bodek et al., 2015), sheep (Letouzey et al., 2015; Emmerson et al., 2019), cow (Cabezas et al., 2014), goat (Tamadon et al., 2017), horse (Cabezas et al., 2018) and non-human primates (Padykula et al., 1989; Wolff et al., 2015) (Table 1). Label-retention, plastic adherence or specific surface markers have been used to isolate endometrial MSC and demonstrate properties similar to human eMSC and bmMSC.

#### Murine Label-Retaining Stromal Fibroblasts

Quiescent stem-like endometrial stromal cells were first identified in mice as bromodeoxyuridine label-retaining cells (LRC), which accounted for 6% of the population after 12 weeks of chase in normal cycling mice (Chan and Gargett, 2006). They were localized adjacent to luminal epithelium, near blood vessels and at the endometrial-myometrial junction, similar to their basalis location in human endometrium (Schwab and Gargett, 2007). Stromal LRCs were both CD31^–^ and CD45^–^, indicating they were neither endothelial cells or leukocytes, and perivascular LRCs were αSMA^+^ (Chan and Gargett, 2006). Approximately 16% expressed estrogen receptor-α (ER-α) and were recruited into cell-cycle following estrogen stimulation indicating their involvement in cyclical endometrial regeneration (Chan and Gargett, 2006; Chan et al., 2012). LRCs were also positive for stem cell markers c-Kit and Oct4 (Cervello et al., 2007). However, stem-cell antigen-1 (Sca-1) was not expressed in stromal LRCs (Chan and Gargett, 2006).

Recently, CD34^+^KLF^+^ endometrial stromal stem/progenitor cells were identified in the perivascular region in the endometrium of a menstruating mouse model (Yin et al., 2019). They expressed the smooth muscle marker, SM22α, and vimentin and upon estrogen stimulation trans-differentiated into gland-like structures lined with E-cadherin-expressing epithelial cells. SUMO-endopeptidase-1 (SENP1)-deletion in SM22α^+^cells induced SUMOylation and activation of ERα which promoted SM22α^+^CD34^+^KLF4^+^ cell proliferation and transdifferentiation into endometrial epithelium via cyclin D1. Mice share ∼85% of protein-coding regions with humans and have 99% genetic similarity (NIH, 2010), their small size and the development of transgenic humanized mice allow their utility as cost-effective models. More research on mouse eMSC isolation and characterizationis warranted.

#### Ovine eMSC

Endometrial stromal fibroblasts have been isolated from Fars native sheep uteri as plastic adherent cells (Ghobadi et al., 2018) or clonogenic cells from Border-Leicester-Marino ewes (Letouzey et al., 2015). Plastic adherent cells expressed *CD73* but not *CD34*, with osteogenic and adipogenic differentiation potential and karyotype stability over four passages. Population doubling times were directly related to the cell seeding density and inversely to age of the ewe (Table 2). Clonogenic stromal cells were enriched using CD271^+^CD49f^–^ surface markers, showing properties similar to bmMSC and human eMSC, with greater clonogenicity, self-renewal by serial cloning compared to CD271^–^CD49f^–^ fibroblasts, and differentiated into adipogenic, myogenic, osteogenic and chondrogenic lineages. Apart from CD271, human MSC markers CD44, CD90, CD140b, and CD146 did not cross react with ovine eMSC. The lack of specific ovine marker antibodies has hampered further surface phenotype characterization. Although in a perivascular location, ovine eMSC unlike their human counterpart, were not located in close apposition to vWF^+^ endothelial cells. Neither did they co-localize with αSMA suggesting they were not pericytes, but rather perivascular adventitial cells, a cell population with similar properties to MSC (Corselli et al., 2012; Crisan et al., 2012). The ability to purify characterized ovine eMSC has enabled the investigation of their use in uro-gynecological disorders such as pelvic organ prolapse (POP) (Letouzey et al., 2015; Emmerson et al., 2019). The domestic ewe has proven a good large animal model because they develop spontaneous POP after vaginal delivery with incidence increasing with parity, similar to women (Couri et al., 2012; Young et al., 2017). Autologous ovine eMSC in a gelatin hydrogel applied onto a polyamide scaffold and implanted into the vagina of the ovine POP model survived for at least 30 days, modulated the inflammatory response, promoting good tissue integration with no postoperative mesh-exposure (Emmerson et al., 2019), one of the main complications associated with human transvaginal mesh (Frankman et al., 2013).

**TABLE 2:** Endometrial MSC and stromal fibroblasts with MSC properties in mammalian species.

Animal	Cell type	Properties	References
Cow	Plastic adherent stromal cells from early and late luteal phase^a^	• Clonogenic, 0.5–1% cloning efficiency, some alkaline phosphatase positive • Fibroblastic morphology. PDT 20–24 h • Uterine tissue: Express *Stat3*, *Cd44, Cd117*, *Oct4*, and *Sox2* but not *Nanog.* Nuclear Oct4 and Sox2. • Primary culture: Express *Oct4* mRNA, and *Sox2* mRNA and protein but not *Nanog.* • Differentiation: Osteocytes and Chondrocytes (late luteal phase).	Cabezas et al., 2014
Goat	Plastic adherent stromal cells	• Fibroblastic morphology. Clonogenic, PDT ∼ 50–53 h in anestrus stage. • Differentiation: Adipocytes, Osteocytes and Chondrocytes • Stable karyotype during culture expansion	Tamadon et al., 2017
Guinea pig	Plastic adherent stromal cells	• Primary culture: Positive for vimentin, ER and PR; negative for cytokeratin. • Proliferation ↑by EGF and insulin in serum free media. • No effect of ovarian hormones in cell growth despite expression of hormone receptors. • Cultured cells secreted ∼30 different proteins.	Chaminadas et al., 1986; Mahfoudi et al., 1992; Ordener et al., 1993
Horse	Plastic adherent stromal cells	• Clonogenic, highly proliferative cells, PDT 46.4 ± 3.4 h. • Differentiation: Osteocytes and Chondrocytes • Express CD29, CD44, CD90, CD105, MHC class-I but not MHC-II and CD45 • Migrate to chemo-stimulant. • Intrauterine infusion of autologous mucin − stromal cells: survived up to 24 h.	Cabezas et al., 2018 Rink et al., 2018
Mouse	Label-retaining cells (LRC) CD34^+^KLF4^+^ stem progenitor cells	• D3 postnatal BrdU labeling, 12 week-chase; 6% BrdU^+^ stromal cells. • Location: Perivascular, endometrial-myometrial junction, beneath luminal epithelium • BrdU^+^ LRC: Some are α-SMA^+^, ERα^+^ (16%) and Ki-67^+^ (12%) in response to exogeneous estrogen. • Some co-express c-Kit and OCT4. Do not express C31, Sca-1 and CD45 • Express SM22α and vimentin. • Proliferate ↑, migrate to injured epithelium and undergo MET upon 17-β estradiol stimulation. • SUMOylation augments ERα transcriptional activity with ↑ proliferation and ↓ apoptosis.	Chan and Gargett, 2006; Chan et al., 2012 Cervello et al., 2007 Yin et al., 2019
Monkey	Plastic adherent stromal cells	• Expanded cells are uncharacterised. • Differentiation: Tyrosine hydrolase-expressing neural-like cells *in vivo* • *In vivo*: ↑ level of dopamine metabolite HVA in PD model.	Wolff et al., 2015
Pig	Plastic adherent stromal cells	• Clonogenic, 0.035% clonal efficiency. • Express *CD29*, *CD44*, *CD144*, *CD105*, *CD140b* and *CD31* and pluripotency genes *NANOG* and *OCT4*. • Differentiation: Osteocytes, Adipocytes and Chondrocytes. • Highly proliferative compared to adipose-derive MSC. • Express CD29, CD73, CD90, and CD105 but not CD34 and CD45. • Wnt pathway activation: ↓large colonies and ↑ small colonies. ↓ MSC surface markers. Osteogenic but not adipogenic differentiation. • Wnt pathway inhibition: Clonal efficiency unchanged, MSC markers ↑. • Higher growth rate than AD-MSC. • Express CD73, CD90, and CD105 but not CD34 and CD45. • CD105^+^ cultured cells differentiation: cardiomyocyte- and insulin-producing β cell-like cells.	Miernik and Karasinski, 2012 Bukowska et al., 2015 Subbarao et al., 2019
Sheep	Plastic adherent stromal cells CD49f^–^CD271^+^ eMSC	• Express *CD73* but not *CD34* and stable karyotype over four passages. • PDT ∼21 h immature and ∼45 h mature ewes. PDT α cell density • Differentiation: Osteocytes, Adipocytes, and Chondrocytes. • Location: adventitia of arterioles and venules. CD271^+^ αSMA^–^ cells (Figure 2I). • Self-renewal by serial cloning *in vitro*, 5.5% cloning efficiency. • High proliferative, PD time 18.7 h. • Differentiation: Osteocytes, Adipocytes, Chondrocytes and Myocytes. • Express human surface markers CD271 but not CD140b, CD44, CD90, and CD146. • Autologous eMSC on gelatin/polyamide mesh survived 30 d in a sheep model of vaginal prolapse. •↑*COL1A1*, *COL3A1*, *FBN5*, *ELN*, and elastin. •↓myofibroblasts and inflammatory response to mesh. •↓ disrupted muscularis and ↑ elastin fibers •↑mesh stiffness, compressive extensions, breaking load and no exposure.	Ghobadi et al., 2018; Letouzey et al., 2015; Emmerson et al., 2019

*^a^ Luteal phase is the progesterone dominant phase of the estrus cycle; AD-MSC, adipose MSC; ER, estrogen receptor; MET, mesenchymal epithelial transition; PD, Parkinson disease; PDT, population doubling time; PR, progesterone receptor.*

#### Non-human Primate Endometrial Stromal Cells

Endometrial-derived stromal cells from the non-human primate, green monkey engrafted and differentiated into neuron-like cells when injected into the striatum of males with 1-methyl-4-phenyl-1,2,3,6-tetrahydropyridine (MPTP) induced Parkinson’s disease (Wolff et al., 2015). The expanded cells used expressed CD140b, CD146, and CD90, but were otherwise not characterized (Wolff et al., 2015), possibly due to lack of suitable antibodies. The differentiated cells expressed tyrosine hydrolase, a rate limiting enzyme for L-DOPA synthesis, proliferated and produced dopamine metabolites indicating their potential use in cell-based therapies (Wolff et al., 2015). Although non-human primates are ideal for simulating human diseases due to their phylogenetic similarity, their utility in research raises ethical, practical and financial issues.

## Menstrual Blood MSC (MenSC)

Menstrual blood is a readily accessible, non-invasive source of large numbers of endometrial stem/stromal cells (MenSC) (Ulrich et al., 2013; Darzi et al., 2016), easily collected using a menstrual cup (Figure 1B) (Musina et al., 2008; Patel et al., 2008; de Carvalho Rodrigues et al., 2012). Menstrual blood contains fragments of shedding endometrial tissue (Figure 1C) which is cultured directly onto plastic similar to bmMSC. Adherent MenSC have typical stromal fibroblast morphology and rapidly propagate with a doubling time of 18–36 h (Meng et al., 2007; Patel et al., 2008). MenSC have higher proliferative capacity, 30–47 PD before senescence, compared to bmMSC, which are generally limited to ∼20 PD (Cui et al., 2007; Allickson et al., 2011). MenSC yield is 2–4-fold higher compared to bmMSC (Alcayaga-Miranda et al., 2015a). As for other adult stem cells, the lifespan of MenSC is relatively short in comparison with human embryonic stem cells (hESC). MenSC only maintain 50% of their telomerase activity at passage 12 compared to hESCs. However, MenSC have more telomerase activity than bmMSC (Patel et al., 2008), perhaps due to the regenerative capacity of the endometrial stroma.

Besides shed endometrial tissue fragments, menstrual fluid also contains peripheral blood. The source of MSC in menstrual fluid could be from bone marrow as well as endometrium. However, peripheral blood contains exceedingly rare bmMSC, as demonstrated by CFU-F analysis. Just 2 CFU-F were found in peripheral blood from 10 patient samples at a frequency of 0–1 CFU-F per 4 × 10^7^ nucleated cells (Kuznetsov et al., 2001). In contrast, menstrual fluid from 18 healthy women contains 600 CFU-F per ml (Alcayaga-Miranda et al., 2015a), suggesting that these CFU-F are derived from the endometrial fragments rather than circulating bone-marrow-derived MSC. Rigorous lineage tracing in chimeric mouse models has also shown that bone marrow stem cells do not contribute to endometrial stromal lineages (Ong et al., 2018). Thus MenSC, comprising mainly endometrial stromal fibroblasts and a small proportion of eMSC found in menstrual fluid are derived from sloughing endometrial tissue rather than non-shedding bone marrow stroma.

### Markers of MenSC

MenSC possess the classic ISCT bmMSC markers and are positive for HLA-ABC, negative for HLA-DR and do not express hematopoietic lineage markers (Cui et al., 2007; Meng et al., 2007; Darzi et al., 2012; Kazemnejad et al., 2012; Khanjani et al., 2014; Khanmohammadi et al., 2014). MenSCs, similar to clonogenic eMSC, differ from bmMSC as they do not express STRO-1 (Cui et al., 2007; Meng et al., 2007; Patel et al., 2008; Schwab et al., 2008; Khanmohammadi et al., 2014). Another difference is that MenSCs highly express cytoplasmic OCT-4 (Borlongan et al., 2010) which is not expressed by eMSC (Gurung et al., 2015; Gurung et al., 2018b) or conventional bmMSC. Several inconsistencies in MenSC phenotype have been observed for pluripotency markers c-KIT and SSEA-4. Some have reported these markers in isolated MenSCs (Patel et al., 2008; Borlongan et al., 2010), while others were negative (Cui et al., 2007; Hida et al., 2008; Musina et al., 2008; Khanjani et al., 2015). Others observed a different pattern of these markers in isolated MenSC (Meng et al., 2007; Musina et al., 2008; Darzi et al., 2012; Kazemnejad et al., 2012) including expression of SSEA-4 and/or NANOG in some c-KIT^+^ cells from cultured menstrual blood (Meng et al., 2007; Patel et al., 2008; Borlongan et al., 2010). Heterogeneity of MenSs cultures may explain these disparities, resulting from differences in menstrual blood sampling day, collection technique and enrichment protocol.

### Mesodermal Lineage Differentiation

MenSC have been induced to differentiate into mesodermal lineages characteristic of bmMSC (Table 3) to satisfy the minimal criteria for MSC. Similar to marker expression, the degree of MenSC differentiation into mesodermal lineages varies and is dependent on the isolation method (Meng et al., 2007; Darzi et al., 2012; Kazemnejad et al., 2012; Khanjani et al., 2014; Khanjani et al., 2015). MenSCs isolated by density gradient centrifugation and plastic adherence have lower capacity to differentiate toward osteoblasts compared to bmMSC using both cytochemical and molecular analyses (Darzi et al., 2012). In contrast, c-KIT^+^ MenSC possessed similar differentiation capacity to bmMSC (Patel et al., 2008), although, Alizarin red alone without quantification was used. It is unknown whether c-KIT-sorted MenSC have osteogenic differentiation ability.

**TABLE 3:** Comparison of *in vitro* differentiation capability of menstrual blood- versus bone marrow-derived mesenchymal stem/stromal cells.

Target lineage	Differentiation medium	Evaluation	Results	References
Chondrocyte	FCS, FGF2, sodium pyruvate, TGFβ3, BMP6, Dex, ITS+1, and ascorbic acid	sGAG Assay, IF, RT-PCR	• High COL2, proteoglycans expression similar to bmMSC • different *COL9A1* and *SOX92A1* to bmMSC	Khanmohammadi et al., 2012
Osteoblast	FCS or HPR, Dex, ascorbic acid, and β-glycerophosphate	Alizarin red, RT-PCR	•↓ mineralization than bmMSC • Comparable *PTHR, OCN* but lower *ALP* in HPR medium compared to bmMSC.	Darzi et al., 2012
Adipocyte	FBS, Dex, rh-Insulin, IBMX, Indomethacin and ATRA (3 sequential protocols)	Oil red staining, RT- PCR	• Lower differentiation capability compared to bmMSC for all protocols	Khanmohammadi et al., 2014
Decidua-like cells	E_2_ and 8-Br-cAMP	FC, IF, RT-PCR	• Markedly higher decidual differentiation than bmMSC	Sugawara et al., 2014
Cardiomyocyte	FBS or serum free medium ± 5-aza, ± FGF2 (2 protocols)	IF, RT-PCR	• Similar ↑ late-stage cardiac markers under 5-aza and FGF2 versus 5-aza alone •↑ *GJA1* and *TNNT2* in MenSCs while ↑ *GJA1* in bmMSC at the same condition	Rahimi et al., 2014, 2018
Hepatocyte	Three-step protocol – Serum free or FBS medium with EGF, FGF2, DEX, ITS+1, NTA, HGF, and OSM	ICC, RT- PCR, functional assays	• Higher *ALB*, *CYP7A1*compared to bmMSC • Express *ALB* and *CK-*18, secrete albumin and produce glycogen similar to bmMSC	Khanjani et al., 2014
Neural	P4-8F with EGF and FGF2 then BDNF, FBS, horse serum, N2 and all-trans- RA Serum-free N2, B27, FGF2, EGF, IBMX, dbcAMP	IF, RT-PCR, EP ICC, BDNF secretion	• Similar ↑ NES, GFAP, but ↑ *GFAP* in bmMSC • Similar ↑ MAP2, GABABR1/2 and TUBB3 •↑ K^+^, Ca^2+^, Na^+^ channel genes with EP recording • Similar ↑ TUBB3, NEUN, and GFAP to bmMSC • Secretion of BDNF in both MenSCs and bmMSC	Azedi et al., 2017 Kozhukharova et al., 2018
Glial lineage	Two-step protocol via NSC: P4- 8F medium with EGF and FGF-2 ATRA, FBS, horse serum, N2 and rh-PDGF for glial cells	IF RT-PCR	• Similar ↑ *OLIG2* and NES in NSCs •↑ GFAP, *OLIG2*, MBP in glial-like cells derived from both MenSCs and bmMSC	Azedi et al., 2014

*5-aza, 5-Azacytidine; 8-Br-cAMP, 8-Bromoadenosine 3′,5′-cyclic adenosine monophosphate; ALB, Albumin; ALP, Alkaline phosphatase; ATRA, all-trans retinoic acid; BDNF, brain-derived neurotrophic factor; FGF2, Basic Fibroblast Growth Factor; BGP, β-glycerophosphate; BMP6, Bone morphogenetic protein 6; bmMSC, Bone Marrow derived mesenchymal stem cells; cAMP, Cyclic adenosine monophosphate; CK-18, cytokeratin 18; CYP7A1, cytochrome P450 7A1; dbcAMP, Dibutyryl Cyclic Adenosine Monophosphate; Dex, Dexamethasone; E2, estrogen; EGF, Epidermal growth factor; EP, Electrophysiology; FCS, Fetal calf serum; FC, Flow cytometry; GABABR, gamma aminobutyric acid type b receptor; GFAP, Glial Fibrillary Acidic Protein; GJA1, Gap junction alpha-1 protein; HGF, Hepatocyte growth factor; HPR, human platelet releasate; IBMX, M3-isobutyl-1-methyl-xanthine; ICC, immunocytochemistry; IF, immunofluorescence; ITS+1, insulin–transferrin–selenium, linoleic-BSA; MAP2, Micro-tubule-associated protein; MBP, Myelin basic protein; MenSCs, Menstrual blood-derived mesenchymal stromal cells; NES, Nestin; NEUN, Neuronal Nuclear Protein; NSC, neurosphere-like cells; NTA, Nitrilotriacetic Acid; OCN, Osteocalcin; OSM, Oncostatin M; RA, Retinoic Acid; rh-PDGF, recombinant human platelet-derived growth factor; RT-PCR, Reverse transcription polymerase chain reaction; sGAG, sulfated Glycosaminoglycan; TGFb3, Transforming growth factor b3; TNNT2, Cardiac muscle troponin T.*

Adipogenic differentiation of MenSC isolated by density gradient centrifugation and plastic adherence is limited when assessed by Oil-red O staining (Musina et al., 2008) and significantly lower than umbilical cord MSC (Jin et al., 2013) and bmMSC (Khanmohammadi et al., 2014). Fortification of adipogenic induction medium with rosiglitazone promoted MenSC differentiation into adipocytes as analyzed by molecular and cytochemical techniques (Khanmohammadi et al., 2014). Purifying cultured MenSC using the c-KIT (CD117) marker resulted in high adipogenic differentiation capacity (60–70%) (Patel et al., 2008), likely due to a more homogeneous MSC population, although it is unknown if CFU-F are also purified in the c-KIT^+^ subpopulation of cultured MenSC.

Differentiation of MenSC into the chondrocytic lineage has been demonstrated for CD117 purified cells in 2D cultures yielding 45% positive cells, similar to bmMSC (Patel et al., 2008). Unfractionated MenSCs cultured in a 3D nanofibrous scaffold enhanced chondrogenic commitment compared to a 2D culture system (Kazemnejad et al., 2012), with extensive cartilage-like extracellular matrix containing ∼50% more glycosaminoglycan than control MenSCs differentiated in 2D. Chondrogenic differentiation requires very low O_2_ tension, which is better established in 3D compared to 2D cultures. MenSCs produce high levels of Activin A, IGF-1, and FGF2, key growth factors involved in chondrogenesis (Ren et al., 2016; Uzieliene et al., 2018).

Co-culture of MenSC with murine cardiomyocytes generated spontaneously beating human cells expressing cardiac specific markers, indicating their differentiation into troponin T-expressing cardiomyocytes (Hida et al., 2008). *In vitro* cardiac differentiation of MenSC was compared with bmMSC using two differentiation protocols and showed that continuous FGF2 was superior to 5-aza-2′-deoxycytidine alone, as shown by increased levels of late-stage cardiac markers (connexin 43 and Troponin T) (Table 3). This suggests that FGF2 has a key role in differentiating cardiac cells from MSC sources. MenSC had greater capacity than bmMSC to differentiate toward cardiomyocytes regardless of the differentiation protocol (Rahimi et al., 2014).

Collectively, the differentiation capability of unfractionated MenSC isolated using conventional methods into two mesodermal lineages (osteoblasts and adipocytes) is lower than bmMSC isolated in a similar manner. This suggests differences in tissue specific MSC properties likely reflecting their tissue of origin. Purification of the rarer perivascular MSC with specific markers appears to improve MenSC differentiation, although c-KIT is not expressed on perivascular cells of human endometrium *in vivo* (Figure 2), but whether induced in cultured perivascular eMSC is unknown.

Endometrial stromal fibroblasts naturally differentiate into decidualized stromal cells *in vivo*. Decidualization is mediated by estrogen and progesterone, and supports the establishment and continuation of pregnancy. MenSC have been differentiated into decidual-like cells with 8-Br-cAMP and progesterone, showing morphological changes and increased expression of the decidualization markers prolactin and insulin-like growth factor binding protein-1 (IGFBP-1), and attenuated expression of MSC surface markers (Sugawara et al., 2014). Greater secretion of these decidual proteins was observed for MenSC compared to bmMSC and amnion MSC (Domnina et al., 2016). It is possible that easily obtained MenSC could be differentiated into decidual-like cells as a potential strategy to mitigate infertility associated with insufficient endometrial decidualization.

### MenSC Differentiation Into Ectodermal and Endodermal Lineages

Cultured MenSC transdifferentiate across lineage boundaries into ectodermal (neurons and glia), and endodermal (hepatocyte) lineages (Table 3). c-KIT sorted MenSCs express neuronal phenotypic markers when grown in appropriately conditioned medium (Borlongan et al., 2010). MenSC formed neurosphere-like cells and then differentiated into neural and glial-like cells comparable to bmMSC, and shown by increased expression of classic neural markers (nestin, microtubule-associated protein 2, gamma-aminobutyric acid type B receptor subunit 1 and 2, and tubulin β3 class III) (Table 3) (Azedi et al., 2014, 2017).

Differentiation of MenSC into hepatocytes is dependent on the concentrations of hepatocyte growth factor (HGF), oncostatin M (OSM) and removal of serum from the induction medium (Khanjani et al., 2015). Up-regulation of albumin and *CYP7A1* expression was higher in MenSC- compared to bmMSC-derived hepatocyte-like cells, although cytokeratin-18 expression, albumin production and glycogen accumulation were either lower or not different (Khanjani et al., 2014), indicating similarity between MenSCs and bmMSC hepatocyte-like cell differentiation potential.

Unfractionated MenSC have potential to differentiate into keratinocyte-like cells, generating epidermal lineage markers via co-culturing with keratinocytes derived from the foreskin of healthy newborns (Faramarzi et al., 2016; Akhavan-Tavakoli et al., 2017). MenSC were also induced to differentiate into keratinocyte-like cells in 3D culture with human foreskin-derived keratinocytes on a bilayer scaffold composed of amniotic membrane and silk fibroin (Arasteh et al., 2018; Fard et al., 2018). The MenSC-derived keratinocytes expressed keratinocyte-specific markers *K14*, p63 and IVL (involucrin). Generating keratinocytes from MenSC on an efficient natural construct has potential applicability for MSC-based skin wound healing and regeneration (Arasteh et al., 2018; Fard et al., 2018), although these tissue engineering constructs have yet to be evaluated in an animal skin wound repair model.

MenSC show considerable capacity to differentiate into numerous lineages *in vitro*, although have lesser ability for several bmMSC lineages. It would now be important to demonstrate the potential of MenSC to undergo differentiation into these lineages *in vivo* in animal models. Genomic sequencing (e.g., RNAseq) would also shed light on how far down the various lineages MenSC differentiate.

## Immunomodulatory Properties of eMSC and MenSC

Since the first report on the immunomodulatory properties of MSC in 2002 (Bartholomew et al., 2002), numerous studies have shown significant impact of MSC on key cells of the innate and adaptive immune systems (Le Blanc and Mougiakakos, 2012; Wang et al., 2014). These non-stem cell properties have been exemplified in bmMSC (da Silva Meirelles et al., 2006). MSC regulate monocyte infiltration to the site of injury, macrophage polarization and dendritic cell maturation (Jiang et al., 2005; Kim and Hematti, 2009). MSC also inhibit NK cell proliferation and target cell killing, and secrete IFN-γ (Sotiropoulou et al., 2006). In the adaptive immune system, MSC suppress T cell proliferation, shifting Th17 cells toward T regulatory cells (Tregs) (Di Nicola et al., 2002; Sato et al., 2007). MSC also impede B cell proliferation (Augello et al., 2005; Corcione et al., 2006). Mechanism of interaction relies on direct cell-cell contact and/or MSC secretion of immunosuppressive factors including Indoleamine 2,3 deoxygenase (IDO), prostaglandin E2 (PGE2), nitric oxide (NO), human leukocyte antigen G5 (HLA-G5), IL-10, IL-6 and TGF-β (Di Nicola et al., 2002; Ren et al., 2010). MSC have also been used to successfully treat patients with severe immune disorders, including Graft-Versus-Host Disease (GVHD) and Crohn’s disease (Dalal et al., 2012). This extensive literature indicates that MSC have immunosuppressive rather than immunostimulatory functions. Here we review the literature on the immunomodulatory properties of eMSC and MenSCs.

### *In vitro* Immunomodulatory Properties of eMSC

The immunomodulatory properties of perivascular eMSC have only recently been investigated. A transcriptional study on CD140b^+^CD146^+^ eMSC revealed that perivascular eMSC differentially expressed several immunomodulatory genes compared to endometrial stromal fibroblasts (Spitzer et al., 2012) (Table 4). A similar gene profile of differentially expressed genes was observed between SUSD2^+^ perivascular eMSC and SUSD2^–^ stromal fibroblasts (Murakami et al., 2014) (Table 4), including anti-inflammatory *IL10*. The gene profile of perivascular eMSC treated and non-treated with the TGFβ receptor (TGFβR) inhibitor, A83-01 to prevent apoptosis and senescence during culture expansion, revealed upregulation of many immune response genes in treated cells, including interleukins (*IL15, IL33, IL6ST*), TNF and IFNγ related genes, PGE2 synthesis genes (*PLA2G4A, PTGS2/COX-2*, *PTGES)*, and Toll-like receptors, *TLR2* and *TLR3* (Gurung et al., 2018b). Together, these finding suggest that eMSC have the ability to interact with the immune system in a similar manner to bmMSC, exerting immunosuppressive functions through paracrine mechanisms and by direct contact with the target cell.

**TABLE 4:** Immunomodulatory properties of endometria MSC, stromal fibroblasts, and MenSC.

Cell type	*In vitro* studies	References	Cell type	*In vivo* studies	References
**Perivascular endometrial mesenchymal stem cells**					
Fresh CD140b^+^CD146^+^ eMSC SUSD2^+^ eMSC, Dec-SUSD2^+^ eMSC SUSD2^+^ eMSC cultured in SM SUSD2^+^ eMSC cultured in A83-01/SFM	•↑ *JAG1*, *TGFβ1*, *CXCR4*, *IL1RAP*, *IL1RL1* • *↔ IDO and PGE2* • *↑ CXCL1*, *CXCL10*, *CCL5*, *IL6*, *IL8* • *↓ IL33*, *AOC3*, *CCL7* eMSC and mouse splenocytes cocultured • Dose dependently ↓ lymphocyte proliferation •↑ FOXP3 Treg in co-culture blocked by TGFβ inhibitor • eMSC produced IL17 and DKK-1 •↑ *SOD3*, *SOD2*, *SOCS3*, *CXCL12*, *CXCL16*, *CCL8*, *IL-15*, *IL33*, *IL6, TLR2/3*, *IL1R1*, *IL6R*, *TLR2*, *TLR3, PLA2G4A*, *COX2*, *PTGES, C3, C2*, *CFB*, *CFD* and *CFI* •↑ CCL2, GM-CSF, IGFBP-2, DKK-1 and EGF. • Secrete IL6, IL8, MCP-1, MIF, MCP-3 and CXCL1.	Spitzer et al., 2012 Murakami et al., 2014 Yang et al., 2019 Gurung et al., 2018b	SUSD2^+^ eMSC in SM	Skin wound repair rodent models implanted with eMSC seeded scaffolds ➢ **Non-Degradable mesh- Rat** • M1 to M2 switch at 30 days •↓CD68^+^ MQ (90 days) ➢ **Non-degradable – NSG and BL6 mice** • C57BL6: ↓ IL1β and TNFα secretion (3d). ↑*Arg, MRC* and *IL10* (3 and 7d). • NSG: ↓ *Arg* (30d), *MRC* (14d). ↓*TNF*α (7 and 30d). ➢ **Degradable Nanofiber – NSG mice** •↑ Cellular infiltration around scaffold •↑M2 MQ and few M1 MQ (qualitatively) • *IL1β, TNFα, CCL2, CCL3,4,5,7,12,19 CXCL1,2,10 –CCR1, CCR7* (1w), ↑ *Arg1, Mrc1*, *IL6* and *Il4ra* (6w). ➢ **3D printed mesh – NSG mice** •↑ M2 MQ and ↓ M1 MQ within implanted biomaterial (1w).	Ulrich et al., 2014a Darzi et al., 2018 Mukherjee et al., 2019b Mukherjee et al., 2020 Paul et al., 2019
**Endometrial stromal cells**					
Plastic adherent ESC	IFN and TNF stimulated ESC for 3 and 7 days • Express IFN-γ1 and TNF I/II receptors •↑ expression: HLA-I but not HLA-II • IDO, IL6 and PGE2 production •↓ contact-induced CD4^+^ T cell proliferation.	Queckborner et al., 2020	Plastic adherent stromal cells	➢ **EAE in BL6 mouse** •↓ EAE score •↓ MN, TH1 and TH17 in CNS •↑ IL10, IL27 and IDO in mouse splenocytes	Peron et al., 2012
**Menstrual blood stem cells**					
Plastic adherent MenSC	Proliferation of allogenic PBMCs in MLR •↓ proliferation at 1:1 and 1:10 (MenSC: PBMC), with higher inhibition at 1:1. Proliferation of allogenic PBMCs in MLR •↓ proliferation at high MenSC concentrations (1:1 or 1:2; MenSC:PBMC ratios) •↑ proliferation at low MenSC concentrations (1:32 or 1:64; MenSC:PBMC ratios) Mo differentiation and maturation • Interfere with Mo ↑ immature DC: ↓CD80, 86, HLA-DR on DCs. • Interfere early stages DC maturation: ↓ CD14, CD1a on DCs. •↑IL6 and IL10 in co-culture with Mo. IL2 supplemented NK cells co-culture with IL1β, IFN ± pre-treated MenSCs • Untreated MenSCs dose dependently ↑ NK cell proliferation • Treated MenSCs ↓ NK cells proliferation. •↓ NK cell cytotoxicity, GzmA, GzmB and perforin. PHA-activated PBMC ± IFNγ and IL1β pre-treated MenSCs. •↓ proliferation at both 1:10 and 1:100 MenSC:PBMC ratios •↓ %CD4/CD8+ IFNγ+ cells •↓ %Th17 cells at 1:10 but not in 1:100 MenSC:PBMC ratio	Wang et al., 2012 Nikoo et al., 2012 Bozorgmehr et al., 2014 Shokri et al., 2019 Luz-Crawford et al., 2016	Plastic adherent MenSC	➢ **DSS induced colitis in BL6 mouse** •↓ intra-colon IL-2 and TNF-α, ↑ IL-4, IL-10. •↓ MHC-II expression in splenic DCs. •↓CD3^+^CD25^+^ and CD3^+^CD8^+^T cells, and ↑ CD4^+^CD25^+^Foxp3^+^ Tregs. ➢ **Anti- PD-L1 ±** **pre-treated MenSCs in DSS induced colitis in mouse.** •↓ infiltration of immune cells. •↓ TNFα and IFNγ in serum and colon. •↓ CD4 and CD8 T cells in colon. •↓CD11c^+^MHC-II^+^ DC in spleen, total MQ but ↑ M2 MQ. •↑CD206 cells and CD4^+^CD25^+^ FOXP3 Treg. ➢ **DSS induced colitis in mouse.** •↓ immature splenic plasma cells and deposition of IgG in the colon. •↑regulatory B cells and IL10. ➢ **CIA and GVHD in mice** • CIA model: ↑ TNF and IL6 • GVHD: ↑ CD45 cells in the spleen ➢ **Allograft heart transplantation in mouse** •↓ IgG and IgM production, and B cells activation.	Cabezas et al., 2014 Shi et al., 2019 Xu et al., 2018 Luz-Crawford et al., 2016 Xu et al., 2017

*CIA, collagen-induced arthritis; Dec, decidualised; DSS, dextran sodium sulfate; EAE, Experimental Autoimmune Encephalomyelitis; FOXP3, Forkhead box 3; Gzm, Granzyme; GVHD, Graft versus host disease; HLA, Human leukocyte antigen; IDO, indole-amin-2, 3 dioxygenase; IFN-γ, Interferone-γ; Ig, Immunoglobulin; IL, Interleukine; MES, Melt electrospinning; MHC-II, Major histocompability antigen class-II; MLR, Mixed leukocyte reaction; Mo, Monocyte; MQ, Macrophage; NK, Natura killer; PBMC, Peripheral blood mononuclear cell; PD-L1, Programmed death-ligand 1; PGE 2, Prostaglandin E2; PHA, Phytohemaglutinin; Th, T helper; TNF-α, Tumor necrosis factor-α; Treg, Regulatory T cells.*

eMSC also influence T cell function by suppressing ConA-stimulated murine T lymphocyte proliferation in a dose-dependent manner (Yang et al., 2019). Blocking the TGFβR in eMSC reversed the immunosuppressive effects of eMSC on T cell proliferation, indicating a role for the TGFβ signaling pathway. Of the various mechanisms assessed, neither IL-10, PGE2, TGFβ or Tregs mediated the eMSC anti-proliferative effects on T cells, however, IL-17A and Dickkopf-1 (DKK-1) were secreted and may be candidates involved. Systemic eMSC failed to inhibit swelling in a T cell-mediated mouse model of skin inflammation, suggesting that eMSC have distinct immunomodulatory properties that may not be sufficient to restrain some T cell-mediated events (Yang et al., 2019).

#### eMSC Effects on Foreign Body Response to Implanted Scaffolds *in vivo*

The immunomodulatory role of perivascular eMSC has been explored in animal models of foreign body response to the implanted biomaterials (Table 4). In a nude rat wound healing model, human eMSC seeded on a gelatin-coated polyamide mesh initially increased inflammatory M1 macrophages at the mesh-tissue interface, followed by reduced inflammation around the mesh filaments by switching macrophages from an M1 to a wound healing M2 phenotype. Long term, there were reduced CD68^+^macrophages at the mesh tissue interface (Ulrich et al., 2014a). Human eMSC seeded on the same polyamide/gelatin mesh inhibited secretion of the inflammatory cytokines IL1β and TNF in early stages of the host response, whereas induced gene expression of anti-inflammatory M2 markers was observed later (Darzi et al., 2018). These responses occurred earlier and were more marked in the immunocompetent than immunocompromised mice. This favorable immunosuppression by eMSC was confirmed subsequently by eMSC-seeded degradable poly(l -lactic acid)-co-poly(ε-caprolactone) (PLACL) nanofibers and 3D bio-printed tissue engineering constructs in the same wound healing immunocompetent mouse models (Mukherjee et al., 2019b; Paul et al., 2019).

### *In vitro* Immunomodulatory Properties of MenSC

The immunomodulatory properties of MenSC have been also been investigated, often in comparison with bmMSC. MenSC effects have been mainly studied on T cell responses. In mixed lymphocyte reactions (MLR), comprising MenSCs mixed with allogenic human peripheral blood mononuclear cells (PBMCs), cellular proliferation, IFN-γ and TNF levels were suppressed, while IL-4 production increased (Murphy et al., 2008). The MenSC suppressive effect on the allogenic MLR was dose-dependent and biphasic, with a high MenSC:PBMC ratio (1/2) suppressing PBMC proliferation and a low ratio (1/32) supporting proliferation (Nikoo et al., 2012). MenSC effects on cytokine levels were similarly concentration dependent with reduced anti-inflammatory IL4^+^IL10^+^CD4^+^ T cells at low MenSC:PBMC ratio compared to bmMSC (Luz-Crawford et al., 2016). These effects suggest distinct immunosuppressive activity of MenSC, similar to eMSC and appear less than bmMSC. This may be due to more HLA-DR molecules on the MenSC surface and fewer IFN-γ receptors, and that MenSC produce less IDO, COX2, and Activin A (Luz-Crawford et al., 2016) compared with bmMSC.

**FIGURE 3:**
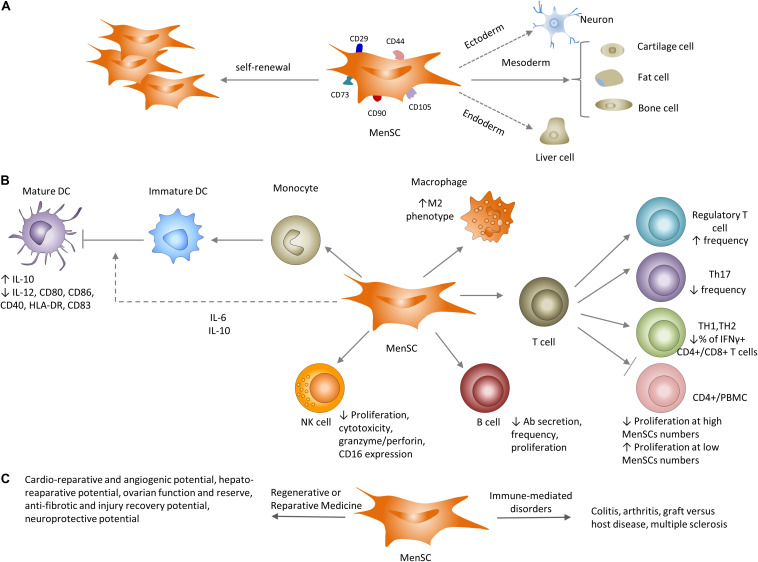
Properties of MenSCs *in vitro* and *in vivo*. **(A)** Similar to their well-known counterparts (bmMSC), MenSCs have the ability to differentiate to cells of different lineages and express typical MSC-associated markers. **(B)** Impact of MenSCs on the adaptive and innate immune systems. MenSCs secrete IL-6 and IL-10 that inhibit optimal maturation of human monocyte-derived dendritic cells (DC). MenSCs shift macrophages toward the M2 (anti-inflammatory) profile. MenSCs either inhibit or support T cell proliferation depending on MenSC/T cell (PBMC) ratio. MenSCs increase Treg frequency, decrease the number of TH17 and IFNγ^+^ CD4^+^/CD8^+^ T cells and cytotoxic capacity of NK cells. MenSCs decrease antigen-specific antibody secretion and proliferation of B cells. **(C)** Utilization of MenSCs in preclinical settings ranges from immune-mediate disorders: arthritis and GVHD to regenerative and reparative applications: cardio protection, liver and lung fibrosis and neuroprotection.

Pretreatment (licensing) of bmMSC with an inflammatory stimulus enhances their immunosuppressive properties. In contrast to the anti-proliferative effect of IFN-γ-pre-treated bmMSC on CD4^+^ T cells, IFN-γ-IFN-g-pre-treated MenSC show a milder response (Aleahmad et al., 2018). Untreated MenSC cocultured with CD4^+^ T cells at ratios of 1:2–1:8 had a pro-inflammatory effect, increasing the proliferation of anti-CD3/CD28-acitivated T cells, and IFN-γ pretreatment only partially reduced this effect, suggesting that IFN-γ responsiveness of MenSC is comparatively lower than bmMSC (Figure 3) (Aleahmad et al., 2018). The impact of MenSC on human T cell proliferation *in vitro* is complex and depends on the cytokine milieu, T cell stimulating factors, MenSC/T cell ratio and the co-culture system. MenSC have a weaker and distinct *in vitro* effect on T cell proliferation compared to bmMSC (Aleahmad et al., 2018), possibly similar to the lesser characterized eMSC responses.

As expected, MenSC also have an impact on the innate immune system, including human blood monocyte-derived dendritic cells (MoDCs) (Table 4) (Bozorgmehr et al., 2014), NK cells and tissue macrophages. MenSC co-cultured with human monocytes interfered with MoDC differentiation at the phenotypic level. The immature DCs expressed suboptimal co-stimulatory molecules and had low levels of CD40, CD80, CD83 and CD86. MenSC secreted IL-6 and IL-10 (Bozorgmehr et al., 2014), which typically inhibit monocyte to DC differentiation (Allavena et al., 1998; Chomarat et al., 2000). Uterine NK (uNK) cells constitute the main component of the endometrial innate immune system, comprising 50–70% of lymphoid cells in early pregnant endometrium (Moffett-King, 2002). uNK cells function in maintaining a successful pregnancy by preventing allorejection of the embryo and regulating vascular remodeling (Hanna et al., 2006; Kalkunte et al., 2009). Dysregulation of uNK cell function may be involved in the pathogenesis of recurrent pregnancy loss (Dosiou and Giudice, 2005) indicating the importance of tightly regulating uNK cell cytotoxic function. Unstimulated MenSCs induced NK cell proliferation partly due to lower production of IGFBPs 1-4 compared to bmMSC. However, in a pro-inflammatory milieu involving IFN-γ/IL-1β, MenSCs substantially inhibited NK cell proliferation through production of IL-6 and TGF-β (Shokri et al., 2019). IFN-γ/IL-1β-stimulated MenSC also curbed NK cell cytotoxicity by decreasing granzyme A, granzyme B, and perforin expression. NK cells also killed MenSC in a MHC- and time-dependent manner. These results suggest a critical role for MenSC in endometrial tissue homeostasis and induction of a pregnancy-friendly phenotype in decidual NK cells.

#### MenSC Immunomodulatory Effects *in vivo*

Recently it was shown that MenSC influence the humoral immune responses in a mouse model of heart transplantation by attenuating antibody responses (Xu et al., 2017). MenSC injection 24 h following allograft heart transplantation prolonged graft survival in recipient mice by rapidly reducing intragraft deposition of donor-specific IgG and IgM antibodies and reducing donor-specific antibody secreting B cells. It remains to be investigated whether MenSC ameliorate autoantibody production in patients with antibody-mediated autoimmune diseases.

In a murine model of colitis, intravenous MenSC injected 2–8 days after disease induction (Cabezas et al., 2014) increased intra-colon IL-4 and IL-10 and decreased IL-2 and TNF, and splenic dendritic cells expressed lower levels of MHC-II compared to untreated controls (Cabezas et al., 2014). The percentage of CD3^+^CD25^+^ and CD3^+^CD8^+^ T cells was reduced and Tregs increased. MenSC also promoted F4/80^+^CD206^+^ M2 macrophage migration and reduced IgG deposition in the injured colon, decreased splenic plasma cells, increased regulatory B cells (Xu et al., 2018), and appeared to mediate these immunomodulatory effects via PD-L1 (Shi et al., 2019).

Not all studies have corroborated the immunomodulatory effects ofMenSC in disease models. MenSC failed to switch T cell-related immune responses toward an anti-inflammatory direction in a murine model of arthritis (Luz-Crawford et al., 2016) (Table 5). MenSC injection increased splenic TNF levels and did not reduce lymph node pro-inflammatory Th17 cells and in contrast, bmMSC did not exert any beneficial impacts on disease progression. MenSC injection into a humanized GVHD mouse model, where irradiated NOD-SCID mice received human PBMC, has been efficacious in reducing disease severity (Luz-Crawford et al., 2016). In this acute inflammatory model, intra-peritoneal MenSC were superior to bmMSC, in contrast to the arthritis model. MenSC improved intestine structure and survival rate. However, immunosuppressive functions were not observed for MenSC, but rather their effect was attributed to their regenerative capacity. MenSCs expressed high levels of VEGF and FGF2, and induced greater vascularization compared with bmMSC. MenSC also had a greater migratory capacity to target organs due to their higher expression of surface CXCR4.

**TABLE 5:** Preclinical and clinical applications of endometrial MSC and Menstrual blood stromal cells.

Disease	Animal model	Findings	References
Perivascular endometrial mesenchymal stem/stromal cells			
Pelvic organ prolapse	• Multiparous sheep, vaginally implanted autologous ovine eMSC seeded PA/G and PA mesh • Rat, SC implantation of DiO-eMSC seeded on PA/G • Mouse, SC implantation of bio-printed eMSC on 3D printed scaffold • NSG mouse, implantation of eMSC seeded degradable electrospun nanofiber.	• 30 days eMSC survival, ↓ myofibroblast, ↑ elastin, ↓ CD45^+^ leukocytes •↑ neovascularization (7d), ↓ mesh/tissue stiffness, crimped collagen deposition • Better tissue integration, collagen deposition • Better tissue integration, ↑Cellular infiltration into mesh	Emmerson et al., 2019 Ulrich et al., 2014a Paul et al., 2019 Mukherjee et al., 2019b
Parkinson disease	• Mouse, immunodeficient and immunocompetent MPTP lesions	• Migrated and differentiated to lesion, ↑ striatal dopamine and its metabolites	Wolff et al., 2011
**Endometrial stromal/stem cells (ESC)**			
Diabetes mellitus	• Immunocompromised mouse Type I diabetes. • ESC differentiated to pancreatic β-like cells were injected into kidney capsule	• Insulin production in single cells from kidney capsule • minimized diabetes-associated complications • reduced blood glucose • prolonged survival of mice	Santamaria et al., 2011 Li et al., 2010
**Menstrual blood stromal/stem cells (MenSCs)**			
Myocardial infarction	• Nude rat, GFP labeled MenSCs injected into the center and margin of infarcted area • Rat, intracardial injection	• Ameliorated left ventricular systolic function • Trans-differentiation to cardiomyocytes •↓ fibrotic areas • Improved cardiac function • trans-differentiation to cardiomyocytes •↑ Akt, Erk1/2, Stat3, proliferation and c-kit^+^ •↓ apoptosis and p38 signaling	Hida et al., 2008 Jiang et al., 2013
Critical limb ischemia	• BALB/c mice, MenSCs injected into hind-limb of CLI mice	• no muscle necrosis in damaged area	Murphy et al., 2008
Acute liver failure (AFL) Liver fibrosis	• BALB/c mouse, IV injection of GFP labeled MenSCs • ICR mouse, IV injection of GFP labeled MenSCs	•↑ Liver regeneration, donor cells homed to injured area, ↓ Ast, Alt, urea and total bilirubin •↑ hepatic markers Alb, Ck18 and Tat • donor cells migrated to injury •↑ liver function, ↓collagen deposition and activated hepatic satellite cells	Fathi-Kazerooni et al., 2017 Chen et al., 2017b
Premature ovarian failure	• C57BL6 Mouse, tail-vein injection of DiO labeled MenSCs Premature ovarian failure models • C57BL6 Mouse, IV injection of MenSCs	•↑ Amh, Fshr, Ki67, ovarian weight, Plasma E2 levels and normal follicles •↓ apoptosis, restored ovarian function and normalized serum ovarian hormones.	Liu et al., 2014; Manshadi et al., 2019 Feng et al., 2019
Poor ovarian response	• POR women, IO injection of MenSCs	•↑ fertilization rate •↑ pregnancy rate	Zafardoust et al., 2020
Acute lung injury	• C57BL6 mouse, IV injection of MenSCs • LPS-induced injury • ICR mouse, IV injection of MenSCs	•↑ pulmonary microvascular permeability, expression of *Pcna*, *Kgf* and *Il-10* •↓ histopathological damage, *Il-1b* expression and Caspase-3 protein expression •↓ inflammatory response and ↑lung tissue repair	Xiang et al., 2017 Ren et al., 2018
Pulmonary fibrosis	• C57BL6 mouse, tail-vein injection of MenSCs bleomycin model	•↓ collagen production, deposition, and wet/dry lung weight	Zhao et al., 2018b
Excisional wound defect	• C57BL6 mouse, intradermal injection of PKH2- labeled MenSCs • Rat, Implantation of amnion seeded with MenSCs	•↑ wound closure, Neovascularization and mature vasculature, collagen content and VEGF • improved wound closure	Cuenca et al., 2018 Farzamfar et al., 2018
Intrauterine adhesion	• ICR mouse, IV injection of DIL labeled MenSCs	•↑ endometrial thickness and microvessel density •↑ conception rate and embryo number	Zhang et al., 2016
Asherman’s syndrome	• Infertile women with severe AS, transvaginal delivery of autologous MenSCS	•↑ endometrial thickness • 2/5 conceived and 1/5 spontaneous pregnancy	Tan et al., 2016
OGD stroke	• Rat, IC and IV injection of MenSCs Neuroprotection model	•↓ behavioral and histological impairments	Borlongan et al., 2010
Sciatic nerve defect	• Rat, MenSCs seeded neural guidance conduit implanted into nerve defect area	• Improved sciatic functional index • Prevented muscle weight- loss •↓ HPLT in MenSCs-seeded conduit group	Farzamfar et al., 2017
Alzheimer’s disease	• Appswe/PSEN1-overexpressed dE9 mouse • IT injection of MenSCs into hippocampus	• Improved spatial learning and memory •↓ plaques and hyperphosphorylation •↑ several Aβ degrading enzymes	Zhao et al., 2018a
Diabetes mellitus	• Mouse type I diabetes, IV injection of MenSCs model of how mice was made diabetic	•↑ insulin production, normalized glucose level •↓Weight loss, prolonged life-span • Recovered islet structure	Wu et al., 2014

*Aβ, Amyloid beta; Alb, Albumin; Alt, Alanine aminotransferase; Amh, Anti-Müllerian hormone; AS, Asherman’s syndrome; Ast, Aspartate transaminase; CD, Cluster of differentiation; CK, Cytokeratin; CLI, Critical limb ischemia; DiO, 3,3′-dioctadecyloxacarbocyanine perchlorate; E2, Estradiol; Fshr, Follicle stimulating hormone receptor; GFP, Green fluorescent protein; HPL, Hot plate latency; IC, Intracerebral; ICR, Imprinting control region; Il, Interleukin; IO, Intraovarian; IT, intrathecal; IV, Intravenous; KGF, Keratinocyte growth factor;, LPS, Lipopolysaccharide; MPTP, 1-methyl-4-phenyl-1,2,3,6-tetrahydropyridine; NSG, NOD scid gamma mouse; OGD, Oxygen glucose deprivation; PA/G, Gelatin coated polyamide; Pcna, Proliferating cell nuclear antigen; POR, Poor ovarian responder; SC, Subcutaneous; TAT, Tyrosine aminotransferase, VEGF, Vascular endothelial growth factor.*

MenSC also have anti-microbial activity. In a cecal ligation and puncture mouse model of sepsis, the combination of MenSC and antibiotic improved the survival rate of affected animals (up to 95%) compared with control animals receiving either treatment alone. The MenSC/antibiotic combination increased bacterial clearance from blood, and reduced the inflammatory cytokines IL-8, TNF, and MCP in peritoneal fluid, without loss of T and B lymphocytes (Alcayaga-Miranda et al., 2015b).

Taken together, eMSC and MenSC have a range of effects on both arms of the innate and specific immune responses (Figure 3), however, the discrepancies between *in vitro* and *in vivo* findings and considerable variation between experimental models necessitate further investigation to identify the underlying mechanisms that orchestrate the cross-talk that MenSC and eMSC utilize to modulate the immune system. Standardization of key variables of human disease and disease models including immune versus non-immune nature of the disease, local versus systemic administration of cells, chronic versus acute disease, and the dose and timing of injected MenSC as well as MSC cell type (perivascular versus unfractionated stromal fibroblast populations) and experimental models as has been promulgated for bmMSC will further increase understanding of the distinct properties of eMSC and MenSC.

## Therapeutic Potential of eMSC and MenSC

### Challenges for MSC-Based Therapies in Regenerative Medicine

Hundreds of clinical trials of various MSC have overall shown underwhelming results in regenerative medicine applications (Prockop et al., 2014). This is due in part to the use of preclinical mouse studies using syngeneic mice, MHC-matched cells and achievable dosing (Galipeau and Sensebe, 2018). Large animal preclinical models are particularly important to trial MSC before going to the clinic to resolve these issues. To date long term engraftment of substantial numbers of infused MSC has not been demonstrated, rather only a small population (2–10%) remain in the days following administration (Dimmeler et al., 2014). Allogeneic MSC will be rapidly removed by the innate immune system, and while autologous cells may prevail, few appear to integrate into tissues. This lack of MSC integration is due to the ischemic or inflammatory environment of the diseased tissue/organ and loss of vascular niches which may be replaced with fibrosis. Aged tissues and chronic disease can also hinder MSC integration (Dimmeler et al., 2014). Despite these challenges, MSC can have dramatic effects through their paracrine actions, appearing to reset the innate immune system and promoting endogenous cellular repair without substantial integration (Caplan, 2016).

### eMSC in Regenerative or Reparative Medicine

Since perivascular eMSC originate from a cyclically regenerating tissue with accompanying angiogenesis (Gargett and Rogers, 2001) they are excellent candidates with therapeutic potential for tissue repair and possibly tissue regeneration. Their pericyte and perivascular identity indicate their specific roles in regulating angiogenesis, inflammation and fibrosis (Thomas et al., 2017), suggesting eMSC are a good candidate for regenerative or reparative medicine.

#### Endometrium and Decidua Regeneration

As mentioned above, human eMSC differentiate into decidual cells. This property has been harnessed for generating a tissue engineered uterus through repopulating decellularized extracellular matrix (ECM) constructs with cells, including endometrial stromal fibroblasts (Fu et al., 2014; Hellstrom et al., 2017; Tiemann et al., 2020). This bioengineering approach has been conducted in decellularized uterus from rat (Miyazaki and Maruyama, 2014; Hellstrom et al., 2016; Kuramoto et al., 2018), pig (Campo et al., 2017) and human (Olalekan et al., 2017). Allogeneic endometrial stromal cells and bmMSC seeded into decellularized rat uteri diffused into the matrix and expressed the stromal marker vimentin and endothelial marker CD31, demarcated by cytokeratin expressing epithelial cells (Miyazaki and Maruyama, 2014). Transplantation of these uterine constructs into partially excised uteri, regenerated endometrial tissue which differentiated into desmin-expressing decidual-cells and achieved pregnancies (Miyazaki and Maruyama, 2014). There was evidence of fetal development, however, no evidence of placentation at the graft site. In another approach to reconstitute native, functional endometrium, a rat GFP^+^ endometrial epithelial cell-sheet was layered on two layers of adherent GFP^+^ endometrial stroma cell-sheets and transplanted into a full thickness endometrial defect in a rat model (Kuramoto et al., 2018). Full thickness endometrium with luminal and glandular epithelium and stromal compartments was generated and pregnancy established on the regenerated endometrium with placentation and fetal heart movements detected. Preliminary results using similar approaches in a larger pig model have commenced (Campo et al., 2017). Using human endometrial Side Population cells including stromal cells demonstrated integration of vimentin^+^ and cytokeratin^+^ cells in the decellularized pig uterus. In an *in vitro* model, endometrial stromal cells, which likely contained a small population of eMSC, repopulated decellularized human endometrial substrates and proliferated in the scaffold (Olalekan et al., 2017). Most importantly, the cells underwent decidual changes upon stimulation with an estrogen and progesterone treatment protocol mimicking the human 28-day menstrual cycle. The infiltrated cells expressed estrogen and progesterone receptors, and secreted prolactin and IGFBP-1, markers of decidualization. These recent approaches indicate the feasibility of using eMSC and endometrial stromal fibroblasts as cell sources for regenerative medicine in uterine biology.

#### Pelvic Organ Prolapse

The potential of eMSC in regenerative or reparative medicine has been explored for a common women’s gynecological disorder, pelvic organ prolapse (Ulrich et al., 2013; Emmerson and Gargett, 2016; Gargett et al., 2019; Mukherjee et al., 2019a). An autologous approach has been investigated and tissue engineering constructs comprising eMSC and novel non-degradable mesh (Ulrich et al., 2012), degradable nanofiber mesh (Mukherjee et al., 2019b), and 3D bioprinted mesh/eMSC (Paul et al., 2019) have been explored in both rodent and large animal ovine models (Emmerson et al., 2019). In these studies, xenogeneic human eMSC delivered on a mesh in a model of subcutaneous wound repair, exerted a paracrine effect. DiO- or mCherry lentiviral-labeled eMSC implanted on a non-degradable polyamide/gelatin composite mesh, were detectable for 1–2 weeks in immunocompromised rats and mice (Ulrich et al., 2014a; Darzi et al., 2018) and up to 3 days in immune intact mice (Darzi et al., 2018). Despite this, the eMSC exerted marked paracrine effects, promoting early neovascularization, an anti-inflammatory response and supporting the deposition of physiological, crimped collagen rather than scar (Edwards et al., 2015). These changes induced by eMSC resulted in a clinically relevant outcome, a reduction in the stiffness of the mesh/tissue complex in the long term (90 days) (Ulrich et al., 2014a). In studies using degradable PLACL nanofiber/gelatin (Mukherjee et al., 2019b) or 3D printed PCL scaffolds (Paul et al., 2019), m-Cherry-labeled eMSC again reduced the foreign body response, slowed mesh degradation and induced endogenous cell influx into the scaffold *in vivo*, thereby promoting tissue repair. These are outcomes of clinically desired responses, particularly for application in pelvic organ prolapse repair surgery.

To more accurately model the translational approach to be used clinically, autologous, adventitial perivascular ovine eMSC (Table 2), labeled with paramagnetic nanoparticles conjugated to FITC were surgically delivered with non-degradable polyamide mesh in an ovine model of vaginal repair (Emmerson et al., 2019). A two-step procedure was required, firstly implanting the mesh followed by separate delivery of the eMSC in a collagen gel onto the mesh and crosslinking with blue light *in situ*, followed by suturing. Approximately 10–20% of the autologous eMSC survived 30 days following implantation. As observed in the rodent models, the eMSC modulated the inflammatory response and reduced myofibroblast accumulation around mesh filaments. Important lessons were learnt using this large animal model, particularly related to the separate delivery of eMSC and mesh. This modification to our protocol prevented one of the major adverse events associated with transvaginal mesh use, mesh exposure, that had resulted in the banning of polypropylene vaginal mesh by the FDA. This could not have been predicted from using mouse models of skin wound repair and shows the importance of closely recapitulating the clinical condition in large animal models before clinical translation of an MSC-based therapy.

#### Other Reparative Applications of eMSC

Human and monkey endometrial stromal fibroblasts transdifferentiated into cells with morphological, chemical and electrical activity of dopaminergic neurons have been investigated in several animal models. They engrafted and migrated to site of lesion in chemically induced mouse and green monkey (Table 2) models of Parkinson disease, differentiated to neuronal-like cells resulting in increased striatal dopamine and dopamine metabolite concentrations (Wolff et al., 2011, 2015). However, further studies on clinical outcomes such as the behavioral studies are warranted. Type 1 diabetes mellitus is a clinical condition which can benefit from islet-based cell transplantation. The plasticity of human endometrial stromal fibroblasts has been utilized to generate insulin secreting cells. These differentiated cells produced human insulin, decreased blood glucose levels and minimized diabetes-associated complications such as weight loss, dehydration, cataracts, delayed wound healing and sedative behavior in a mouse model of diabetes mellitus (Li et al., 2010; Santamaria et al., 2011). These differentiated cells were more efficient in a 3D construct, and were resistant to oxidative stress, normalized glucose levels and prolonged survival of the recipient mice (Li et al., 2010).

Both eMSC and endometrial fibroblasts show potential in regenerative and reparative medicine, with early studies suggesting capability in tissue engineering approaches using decellularized tissues or biomaterial scaffolds to deliver the cells to tissues requiring regeneration. Mode of action may be both cellular integration and paracrine.

### MenSC in Regenerative or Reparative Medicine

MenSC are also an attractive cell source with potential for clinical application. MenSC have been assessed in various preclinical animal models of disease and in regenerative and reparative medicine (Table 5). Here, we will briefly review these studies.

#### Cardio-Reparative Potential

One of the first studies proposing the prospective use of MenSC differentiation in the clinical setting assessed the ability of MenSC to improve cardiac function after myocardial infarction. MenSC transplanted into a rat model ameliorated left ventricular systolic function and diminished fibrotic areas (Hida et al., 2008). *In vivo* differentiation of MenSC to cardiomyocytes in infarcted areas was demonstrated in the absence of recipient and donor cell fusion. Similar outcomes were obtained following MenSC injection into the ischemic zones of an immunocompetent rat model of myocardial infarction (Jiang et al., 2013). The therapy resulted in significant preservation of myocardial viability in the infarct zone and improvement in cardiac function, effects mostly attributed to the paracrine activity of MenSCs rather than transdifferentiation to myocardial cells.

#### Hepato-Reparative Potential

MenSC’s ability to differentiate toward hepatic cells *in vitro* (Khanjani et al., 2015) raised the possibility of MenSC application for treating liver disorders. Both MenSC and bmMSC, with the capacity to differentiate toward hepatocytes, prolonged the survival of mice with acute liver failure. MenSC localized in the damaged liver within 2 h following transplantation. This treatment also improved liver histology and architecture within weeks after MenSC injection. The MenSC therapy reduced serum levels of liver enzymes and metabolites (AST, ALT, urea, and total bilirubin) in the mice. Reduced hepatic degeneration, inflammatory cell infiltrate and collagen fiber deposition and improved glycogen storage were observed following MenSC administration (Fathi-Kazerooni et al., 2017), indicating the protective and reparative role of MenSC in acute liver damage.

It is likely that MenSC improvement of liver function is due to paracrine activity rather than differentiating into hepatocyte-like cells in improving liver fibrosis. In a murine model (Chen et al., 2017b), few MenSC differentiated into hepatocyte-like cells, suggesting the complexity of the *in vivo* milieu influencing MenSC differentiation. MenSC migrated to the fibrotic area, reduced already deposited collagen and significantly improved liver function. Nevertheless, end-stage liver fibrosis with few healthy hepatocytes may be a poor environment for survival of MenSC that also diminishes their ability to potentially differentiate into hepatocyte-like cells. This questions the utility of MenSC as a therapeutic modality to patients with cirrhosis. Further investigation in first reducing fibrosis in cirrhosis using MenSCs before attempting to regenerate hepatic tissue is warranted.

#### Ovarian Function and Reserve

The reparative capacities of MenSCs was exploited to treat premature ovarian failure (POF) in mouse/rat models (Liu et al., 2014; Feng et al., 2019; Manshadi et al., 2019; Yan et al., 2019). Intra-ovarian or intravenous injection of MenSCs reduced apoptosis and restored ovarian function, shown by expression of the ovarian markers, follicle-stimulating hormone receptor (Fshr), inhibin α/β, anti-Müllerian hormone (Amh), Ddx4 and Vegfa, and rising plasma levels of the ovarian hormones, estrogen and progesterone. Increased numbers of primary, mature and total ovarian follicle numbers were observed. DiI-labeled MenSC localized to GCs of immature ovarian follicles (Manshadi et al., 2019). Microarray analysis revealed greater similarity between mRNA expression patterns in the ovarian cells posttransplantation and human ovarian tissue than the pre-transplantation pattern in MenSCs, suggesting transdifferentiation into ovarian cells or fusion of MenSCs with murine cells (Liu et al., 2014). More importantly, mated mice grafted with MenSCs had more live births indicating the potential of MenSC to repair ovarian function (Feng et al., 2019). Mechanistic analyses revealed that mice treated with MenSCs improved ovarian microenvironment homeostasis through regulation of the ECM-dependent FAK/AKT signaling pathway (Feng et al., 2019). Similarly, *in vitro*, MenSC increased indices of mouse follicular growth, including survival rate, diameter and antrum formation, *Bmp15* and *Gdf9* expression and maturation rate in a 3D culture system. Secreted progesterone and estradiol increased in co-cultured murine preantral follicles and human MenSC implying a supportive role of MenSCs in follicle development, growth and maturation (Rajabi et al., 2018). In a recent clinical trial in 15 women, intraovarian injection of autologous MenSC in poor ovarian responders increased clinical pregnancy and live births (Zafardoust et al., 2020).

Collectively, MenSC impact on ovarian follicle development through paracrine signaling and potentially transdifferentiation highlights the possible utility of MenSC for ovarian function restoration in patients with POI and also fertility preservation approaches using *in vitro* follicle maturation. These pre-clinical findings and an early phase clinical trial warrant further investigation of the efficacy of MenSC for treatment of POI.

#### Lung Injury

MenSCs have been used to treat LPS-induced acute lung injury in a murine model (Xiang et al., 2017). Intravenously administered MenSC localized to the injured area and demonstrated repair of lung tissue morphology, improved lung microvascular permeability, and reduced clinical symptoms. MenSC exerted an anti-inflammatory effect, reducing lung inflammatory cells and IL-1β, whereas IL-10 was increased in both lung tissue and broncho-alveolar lavage fluid. Keratinocyte growth factor, which plays a major part in repair of damaged lung was increased, and together with decreased caspase-3 expression further supported the protective impact of the infused MenSC. It is likely that the reparative effect of MenSC can be ascribed to cell-cell contact and/or their paracrine function. Similarly, MenSC injection improved acute lung injury scores through normalization of lung O_2_ pressure. MenSC reduced neutrophil frequency, myeloperoxidase activity, and pro-inflammatory cytokines levels in bronchoalveolar fluid (Ren et al., 2018). MenSC injection also reduced inflammation and collagen deposition in a mouse model of bleomycin-induced pulmonary fibrosis (Zhao et al., 2018b). In summary, in acute lung injury and pulmonary fibrosis models, MenSC suppressed innate immune cells, lowered pro-inflammatory cytokines and increased IL-10 in lung, and lung function and morphology were restored, effects likely mediated by paracrine mechanisms.

#### Skin Wound Repair

The reparative potential of MenSCs in a murine excisional wound splinting model (Cuenca et al., 2018) showed that intradermal injection of MenSCs into a persistent wound improved healing and increased angiogenesis. Two pro-angiogenic genes, *Il-8* and *Vegf* were upregulated in the wounds and there was maturation of wound vasculature. The MenSC-injected group also showed high density, and well-organized collagen fibers, suggesting MenSC have potential application for wound healing and cutaneous regeneration. MenSC seeded on an amniotic membrane improved wound closure in a rat excisional wound defect model (Farzamfar et al., 2018). *In vitro*, MenSCs differentiated into keratinocyte-like cells (Akhavan-Tavakoli et al., 2017), an effect potentiated in 3D culture (Fard et al., 2018) or in a biomimetic nanofibrous scaffold, two approaches that increased the wound healing capacity of MenSC (Arasteh et al., 2018). Such data imply the importance of ECM in the differentiation of MenSC into keratinocyte-like cells and further highlight the value of MenSCs as a potential therapeutic in clinical settings.

#### Intrauterine Adhesions and Endometrial Decidualization

The reparative potential of MenSC has been explored in a mouse model of endometrial injury and intrauterine adhesion (Zhang et al., 2016), where the endometrium is partially replaced by fibrotic scar tissue (Gargett and Ye, 2012). Intravascular injection of MenSCs rapidly repaired the injury by increasing microvessel density, resulting in increased endometrial thickness. Fertility was restored with a higher conception rate and larger numbers of implanted embryos compared to untreated controls. MenSC-conditioned medium promoted angiogenesis *in vitro* by activating Akt and Erk pathways and overexpression of genes involved in angiogenesis. Autologous cultured MenSCs administered to 7 women with severe Asherman’s syndrome, where intrauterine adhesions have replaced the endometrium, increased endometrial thickness to 7 mm in five of seven patients (Tan et al., 2016). Of the four patients who had undergone embryo transfer, two conceived and one had a spontaneous pregnancy, suggesting that MenSC reduced fibrosis and promoted functional endometrial repair of the injured endometrium.

#### Neuroprotective Potential

The application of MenSC, which can differentiate toward glia and neural cells *in vitro* (Table 3), for neuroprotection is an emerging research area. In an adult rat stroke model, intracerebral or intravenous MenSC transplantation ameliorated motor and behavioral symptoms and diminished neuronal cell death (Borlongan et al., 2010). Implantation of MenSC-seeded gelatin-based scaffolds in rats with sciatic nerve defect improved sciatic nerve function and reduced and gastrocnemius muscle loss to a similar level as bridging the nerve defect with autologous resected nerve segment (Farzamfar et al., 2017). MenSC also improved cognition function and memory defects in a mouse APP/PS1 model of Alzheimer’s disease (Zhao et al., 2018a). In the hippocampus, more activated microglia were observed which had altered function as less TNF and IL-1β were produced. These activated microglia had higher expression of insulin degrading enzyme and neprilysin, proteases responsible for Aβ plaque degradation. These results highlight the potential of MenSC in improvement of the pathological and cognitive defects in pre-clinical models of Alzheimer disease.

#### Other Potential Reparative Applications of MenSC

The regenerative ability of MenSC to promote muscle regeneration was demonstrated in a murine Mdx model of Duchenne muscular dystrophy, where skeletal muscles degenerate from lack of dystrophin. GFP-labeled MenSC were injected intramuscularly, where they fused with murine myoctes and restored dystrophin in the sarcolemma of muscle fibers (Cui et al., 2007). Similarly, MenSC fuse with myocytes in co-culture and subsequently express dystrophin.

MenSC can stimulate the regeneration of pancreatic islet β cells in a murine model (Wu et al., 2014). MenSC transplantation enhanced differentiation of endogenous endocrine progenitor cells into β-cells resulting in more normal islet morphology and structure, with improved hyperglycemia, glucose tolerance, insulin levels, body weight and survival rate. Although not investigated inflammation reduced by MenSC may have also contributed to the observed results.

In summary, MenSC show potential in regenerative medicine, with early studies suggesting capacity to influence tissue regeneration by paracrine mechanism, fusion and enhancement of endogenous tissue stem cell function.

#### Extracellular Vesicles as a Key Player of MenSC Reparative Function

Increasingly, the effects of MSC are attributed to their ability to secret extracellular vesicles (EVs), including exosomes (Murray and Krasnodembskaya, 2019). MenSCs also produce functionally active EVs that are homogeneous in size (30–170 nM) and express CD81, CD63, and TSG101 (Dalirfardouei et al., 2018). MenSC-EVs contain 895 proteins involved in complement activation, antigen processing and presentation, regulation of adaptive and innate immune responses, apoptosis control and signaling pathways (Marinaro et al., 2019). Licensing MenSCs with pro-inflammatory cytokines such as IFN-γ modulates EV protein cargo, increasing proteins involved in antigen processing and presentation, and miRNA content. MenSC-derived EVs also express high levels of ICAM-1, angiopoetin-2, angiogenin, osteoprotegerin, IL-6, and IL-8. In a mouse model of fulminant hepatic failure, pre-treatment with MenSC-exosomes showed higher survival rates, with reduced serum liver enzymes and pro-inflammatory cytokines, and well-organized hepatic structure (Chen et al., 2017a). Hepatocyte apoptosis and proliferation of liver macrophages were reduced. In a rat model of diabetes, MenSC-derived EVs were tracked in, and enhanced the number of pancreatic β cell islets and raised serum insulin, without impact on non-fasting blood glucose (Mahdipour et al., 2019). MenSC-derived exosomes impacted wound healing in a mouse model of diabetes through M2 macrophage polarization, induction of neoangiogenesis and re-epithelialization (Dalirfardouei et al., 2019).

In summary, the beneficial impacts of MenSC treatment are mostly attributed to the anti-inflammatory properties of MenSC-derived EVs. In some settings the modulatory effects of EVs were superior to the use of intact MenSC. Therapeutic utilization of MenSC-EVs might be advantageous over whole cells by improving the therapeutic index, by introduction of less protein, avoidance of allo- and xenogeneic reactions and their off-the-shelf potential. Nonetheless, their nano-scale size results in rapid clearance from the body necessitating repeated administration in large quantities, although therapy targeted to damaged tissue may overcome this potential challenge. Taken together, MenSC may qualify as a promising therapeutic cell type for future clinical applications. Nevertheless, as with any other treatment modality, the safety of MenSC in the clinical settings needs addressing.

## Clinical Translation of eMSC and MenSC

There are hundreds of clinical trials using MSC either in progress or completed and thousands of patients have been treated. Apart from several spectacular successes the overall results of these clinical trials have been underwhelming (Prockop et al., 2014). MSC were rushed to the clinic before many issues were resolved, including their mechanism of action and the heterogeneity of MSC products. Heterogeneity arises from variability in isolation methods and during culture expansion (Prockop et al., 2014). Thawing cryostored MSC induces cell injury lasting up to 24 h (Moll et al., 2016), suggesting a lack of fitness for purpose of culture-expanded, thawed MSC (Galipeau and Sensebe, 2018). There is also a need for potency assays based on MSC mechanism of action *in vivo* (Prockop et al., 2014; Galipeau and Sensebe, 2018). These are important considerations for investigators seeking to translate eMSC and MenSC as cell-based therapies.

### Serum Free Culture Protocols

The clinical translation of human MSC depends upon largescale production of a homogeneous cell population that is safe and reproducible. Factors contributing to a heterogeneous population are isolation protocol, culture medium and culture environment. The discovery of a single surface marker for human eMSC, SUSD2, has enabled relatively easy purification of a homogeneous starting population. Choice of culture medium and environment majorly influence the generation of large scale homogeneous eMSC and MSC from other sources utilized for clinical use. Until recently, MSC including eMSC have been cultured in fetal calf serum (FCS) containing medium in 20% O_2_ environment (Schwab and Gargett, 2007). FCS provides extracellular matrix, growth factors, hormones and many other nutrients promoting cell grow and proliferation (Cantor, 2019). However, FCS is not a defined source of nutrients with batch to batch variation and presence of unknown components that contribute to heterogeneity (Shahdadfar et al., 2005). Some FCS components induce differentiation, reducing the population of potent MSC (Shahdadfar et al., 2005; Labome, 2012). The use of FCS for *in vitro* MSC expansion also carries a rare risk of xeno-immunization and zoonotic transmission (Hawkes, 2015). Defined medium devoid of FCS is required to produce homogeneous, undifferentiated potent MSC using good manufacturing practices (GMP) for safe use in humans. Replacing FCS with human products including human serum, platelet-poor or -rich plasma and platelet lysate have been used (Dessels et al., 2016). Although more physiological, these alternatives have similar composition variability as FCS and may also induce differentiation, resulting in inconsistent, non-reproducible cell products. Gene profiling of primary CD140b^+^CD146^+^ eMSC showed distinct differences to endometrial stromal fibroblasts (CD140b^+^CD146 ^–^) (Spitzer et al., 2012) and extensive eMSC cultivation in FCS medium in 20% O_2_ led down-regulation of 81% of eMSC-related genes and up-regulation of 55% of fibroblast-associated genes, verifying culture-induced spontaneous differentiation and reduced functionality (Barragan et al., 2016; Gargett and Gurung, 2016). A defined medium and physiological O_2_ environment that generates homogeneous undifferentiated eMSC which are efficacious, safe and reproducible under cGMP guidelines would be appropriate for clinical applications such as pelvic organ prolapse and Asherman’s syndrome.

While various serum-free media are available for culturing human MSC, vast research shows that “one size does not fit all,” indicting the need to identify the specific niche environment for eMSC. Indeed, eMSC attachment and growth were best supported by a fibronectin matrix in xeno-free DMEM supplemented with FGF2 and EGF (SFM) in 5% O_2_, compared to other serum-free commercial media, Lonza-TP-SF and Stem Pro-XF giving similar growth rates to serum-containing medium (Rajaraman et al., 2013). Despite this, like all MSC, the expanded SUSD2^+^ eMSC spontaneously differentiated to non-clonogenic stromal fibroblasts with loss of the eMSC surface markers SUSD2, CD140b, and CD146 indicating cellular heterogeneity and decreased potency (Gurung et al., 2018b).

### Small Molecules to Maintain Undifferentiated eMSC State

Understanding the intrinsic signaling pathways involved in stem-cell fate, self-renewal, proliferation and differentiation, and manipulating them using chemical approaches enables generation of homogeneous potent cells for cell-based therapies (Xu et al., 2008; Li and Ding, 2010). Chemically defined media needs optimization for each cell type using relative cell-growth as a guide. Augmenting SFM with a small molecule (A83-01) targeting the transforming growth factor-β1 receptor (TGFβR) signaling pathway involved in cellular differentiation and enhanced cell growth, mitigated loss of undifferentiated, clonogenic eMSC, overcoming this major bottleneck for clinical translation of eMSC (Gurung et al., 2015, 2018b). A83-01 maintained ISCT properties of eMSC, promoted proliferation of homogeneous SUSD2^+^ eMSC and prevented apoptosis and senescence. Correlation between SUSD2 expression and TGFβ-induced senescence and cell death was also demonstrated in cancer cells using small interfering RNA and TGFβ (Zhang et al., 2017). The potency of A83-01-treated eMSC was further validated by transcriptome profiling, providing insight into the biological nature of eMSC and the probable therapeutic mode of action (Gurung et al., 2015, 2018b). The complexity of TGFβR signaling in eMSC was defined through identification of ∼1200 differentially regulated genes by A83-01 involved in anti-inflammatory responses, angiogenesis, cell migration and proliferation, collagen fibril and extracellular matrix organization, anti-fibrosis and anti-apoptosis (Gurung et al., 2018b). The potency of A83-01-treated eMSC was established by demonstrating increased expression of recently described bmMSC potency genes; *TWIST1, TWIST2, JAG1, LIFR*, and *SLIT2* (Samsonraj et al., 2015; Gurung et al., 2018b). These and our previous findings also highlighted the need for additional surface markers to identify potent MSC and for specific tissues (Rajaraman et al., 2013; Gurung et al., 2015, 2018b; Samsonraj et al., 2015).

The reparative and regenerative capacity of MSC is mainly driven by the secretome or EVs that promote angiogenesis, ECM regulation, immunoregulation and antimicrobial activity. Transcriptional profiling of A83-01-treated eMSC revealed important cellular secretome-regulating genes involved in angiogenesis, anti-fibrosis and immunomodulation; *HGF, VCAM1, PGF, HPSE, SOD3, SOD2*, and *SOD1*. Secretome analysis of the conditioned medium supported transcriptional findings with increased HGF, PTX3, CCL2, IGFBP-2, CCL2, GM-CSF, IGFBP-2, DKK-1, and EGF and decreased THBS1, EMMPRIN, and OPN. HLA-G, a nonclassical MHC-I molecule, and immunomodulatory cytokine functioning at the maternal-fetal interface and some immune privileged adult tissues, was ubiquitously expressed in A83-01-treated eMSC, together with *HLA-A-C* and *HLA-E* and *F*, but not *HLA-II*, an indicator of good tolerance for allogeneic transplantation. HLA-G has been identified in several MSC types (Selmani et al., 2008; Yang et al., 2012; Naji et al., 2013; Ding et al., 2016), and together with other MHC-I molecules correlates directly to their immunosuppressive effect (Yang et al., 2012). These molecules are surrogate prognostic factors used to monitor disease progression or efficacy of transplantation (Nasef et al., 2007). Likewise, RNA-sequencing revealed the first evidence of the ability of A83-01-treated eMSC to secrete exosomes (Gurung et al., 2018b), that may function in their paracrine action. Further transcriptome analysis showed evidence of the antimicrobial potential of A83-01-treated eMSC partially through the secretion of antimicrobial peptides (AMPs), chromogranin B, liver enriched AMP-2 and secretogranin II (Gurung et al., 2018b). Taken together, these findings signify the clinical potential of eMSC and open avenues to examine their use in regenerative medicine as an “off the shelf” as well as cell-derived therapy.

### Safety of eMSC

Spontaneous transformation of MSC is a potential concern for therapeutics, although thorough investigations of earlier studies showed cellular-cross contamination. To date, no cancer due to primary-culture expanded MSC, has been diagnosed in human clinical trials. Nonetheless, the risk of such potential events should be investigated by utilizing surrogate assays, such as telomere length, pluripotency genes, tumor formation, genomic instability, karyotyping and DNA damage response through transcriptomics and *in vitro* and *in vivo* assays. A83-01-expanded eMSC promote telomere stability through *TERC* (Telomerase RNA Component), *TERF1* and *2* (Telomeric Repeat Binding Factors 1 and 2), *TINF2* (TERF1 Interacting Nuclear Factor 2), *TERF2IP* (TERF2 Interacting Protein), *TNKS* (Tankyrase) and *POT1* (Protection of Telomere 1) which collaborate to regulate telomere length and protect cells from chromosomal damage (Gurung et al., 2018b). Using our isolation and expansion protocol, SUSD2^+^ eMSC do not express pluripotency-associated genes, unlike hESC and iPSCs with unlimited growth and tumorigenic potential (Gurung et al., 2015; Gurung et al., 2018b). Although SFM/5%O_2_/A83-01 culture-expanded eMSC survived longer *in vivo* than culture in FCS/20%O_2_ medium, their lifespan was limited and tumors were not detected (Gurung et al., 2018a). Considerable progress has been made in developing expansion protocols for perivascular eMSC showing promise in their safety profile.

### Safety of MenSC

#### Donor Age

Age of donor affects many of MSC functions; cytokine secretion, paracrine activity, anti-apoptotic mechanisms, hematopoietic stem cell supporting capacity and proliferation rate, all of which reduce with aging (Alt et al., 2012). However, the proliferative capacity of MenSC does not appear donor-dependent in individuals aged up to 40 years, but MenSC from older donors had a weaker potential for long-term passaging (Chen et al., 2015).

#### Passage Number

Menstrual blood can be processed up to 72 h after collection without significant change in properties of the plastic adherent MenSC (Liu et al., 2018). As mentioned, culture expansion of MSC is required to obtain enough cells for preclinical or clinical applications. The proliferation rate of MenSC gradually decreased with increasing passage number (Chen et al., 2015), with highly passaged cells increasing in size, losing fibroblastic morphology and appearing senescent. Important signaling molecules, including MAPK, molecules involved in carcinogenesis (PPAR and P53) and immune responses changed with MenSC passage number. Passaging induces aging of MenSC and alters genes involved in transcriptional regulation, stress response, cell proliferation, development and apoptosis, and karyotype at high passages (P20). MenSC underwent aging after 45 PD (Zemel’ko et al., 2011), but even after 68 PDs of a single MenSC sample, maintained a normal karyotype and did not develop tumors (Meng et al., 2007). Multiplex ligation-dependent probe amplification of MenSC genomic DNA at passages two and twelve showed that MenSC maintained a diploid phenotype without chromosomal aberrations (Khanmohammadi et al., 2014), consistent with other reports of a normal female karyotype without chromosomal aberrations using standard cytogenetics at passage 12 (Cui et al., 2007; Patel et al., 2008). Pluripotency protein expression reduced during passaging of MenSCs, from 97.5% OCT-4^+^ cells at the first passage to 19.4% at passage twelve (Khanmohammadi et al., 2014), important for clinical translation. These observations warrant standardization of MenSC isolation and culture protocols for clinical applications.

#### Safety of MenSC

The risk of tumorigenicity, toxicity, autoimmunity, and/or endometriosis following MenSCs injection are crucial safety issues to be established before using MenSC in the clinic. Angiogenic activity of MenSC, raises concern of promoting tumor progression. In a C6 model of rat glioma MenSCs not only failed to stimulate, but also inhibited tumor growth (Han et al., 2009). MenSCs showed no tumor formation in a xenograft rat model of brain stroke with no concurrent immunosuppression (Borlongan et al., 2010). Several acute and chronic tumorigenicity/toxicity experiments in immunocompromised mice using MenSC in clinically relevant doses showed no toxic effects on body and organ weight, biochemistry following necropsy or histopathological changes at injection sites (Bockeria et al., 2013). Concerns of ectopic bone formation on clinical application of bmMSC, are mitigated by the weak osteogenic differentiation capacity of MenSC and could be advantageous for clinical translation.

The next step prior to clinical development of MenSC is to undertake cell tracking of reliably labeled MenSC *in vivo* and determining their mechanism of action. Also important is the development of relevant large animal pre-clinical models of the targeted disease for evaluating MenSC, to improve the likelihood of translating rodent studies into clinical practice. In 2008, four multiple sclerosis patients were injected both intrathecally and intravenously with *in vitro* expanded allogeneic MenSC. Functional and radiological evaluations demonstrated no disease status progression and none of the treated patients showed allergic reactions or ectopic tissue formation (Zhong et al., 2009). No adverse effects were observed in the patients 4 years later in the last reported follow up (Bockeria et al., 2013). Clearly more investigation is required to determine the safety of MenSC for clinical translation.

Identification of eMSC potency makers and serum-free expansion media have enabled gene sequencing of the primary cells and their expanded progeny (Spitzer et al., 2012; Barragan et al., 2016; Gurung et al., 2018b). Comprehensive RNA-seq analysis of SFM/5%O2/A83-01-treated eMSC described the gene expression profiles and uncovered vast range of regulatory pathways which not only aided in understating their mechanism of action but also suggested their potential for broader applications. Much progress are made in understanding the *in vitro* and *in vivo* roles of eMSC, setting a strong foundation for clinical application. Further research to understand and validate eMSC’s potential through careful titration of assays and selection of animal models relative to their potential use is vital.

## Summary

eMSC and MenSC have fulfilled the ISCT criteria for MSC, but thus far, no comparative study has been performed to delineate the potential link between these two MSC types. Nonetheless, considering that eMSC comprise a small cell population residing around endometrial blood vessels while MenSC represent majority of endometrial stromal cells, it seems that the latter is more heterogeneous. Although multi-differentiation capacity of eMSC and MenSC has been documented *in vitro*, the reparative properties of these cells *in vivo* seem to stem from their paracrine rather than differentiation capacity and integration into tissue. Akin to MSC from other sources, MenSC and eMSC have profound immunomodulatory effects and attenuate inflammation, although there are distinct differences highlighting their unique tissue of origin. eMSC and MenSC have been evaluated in numerous preclinical animal models of disease showing substantial angiogenic, anti-fibrotic and anti-inflammatory effects although it is too early to draw a firm conclusion on the same impacts in clinical settings. Culture protocols have been developed that maintain perivascular eMSC in the undifferentiated state during culture expansion, which now need testing in animal models to determine if this more homogeneous MSC product further improves *in vivo* angiogenesis, antifibrosis, anti-inflammatory and prohealing effects. Both eMSC, easily procured without anesthetic, and non-invasively obtained MenSC, imbued with both reparative and immunomodulatory properties are likely promising therapeutic cell types for future clinical applications. Nevertheless, multiple variables need to be standardized particularly heterogeneity of MenSC and the safety and therapeutic potency of eMSC and MenSC in clinical settings needs to be extensively addressed.

## Author Contributions

CG and A-HZ contributed to the conception and review design and revised the manuscript. MB, SG, SD, SN, SK, A-HZ, and CG wrote sections of the manuscript. All authors contributed to the article and approved the submitted version.

## Conflict of Interest

The authors declare that the research was conducted in the absence of any commercial or financial relationships that could be construed as a potential conflict of interest.
